# The interplay between gaze and consistency in scene viewing: Evidence from visual search by young and older adults

**DOI:** 10.3758/s13414-021-02242-z

**Published:** 2021-03-21

**Authors:** Eunice G. Fernandes, Louise H. Phillips, Gillian Slessor, Benjamin W. Tatler

**Affiliations:** School of Psychology, University of Aberdeen, Aberdeen, Scotland AB24 3FX UK

**Keywords:** Scene viewing, Visual search, Gaze following, Semantic consistency, Ageing

## Abstract

Searching for an object in a complex scene is influenced by high-level factors such as how much the item would be expected in that setting (semantic consistency). There is also evidence that a person gazing at an object directs our attention towards it. However, there has been little previous research that has helped to understand how we integrate top-down cues such as semantic consistency and gaze to direct attention when searching for an object. Also, there are separate lines of evidence to suggest that older adults may be more influenced by semantic factors and less by gaze cues compared to younger counterparts, but this has not been investigated before in an integrated task. In the current study we analysed eye-movements of 34 younger and 30 older adults as they searched for a target object in complex visual scenes. Younger adults were influenced by semantic consistency in their attention to objects, but were more influenced by gaze cues. In contrast, older adults were more guided by semantic consistency in directing their attention, and showed less influence from gaze cues. These age differences in use of high-level cues were apparent early in processing (time to first fixation and probability of immediate fixation) but not in later processing (total time looking at objects and time to make a response). Overall, this pattern of findings indicates that people are influenced by both social cues and prior expectations when processing a complex scene, and the relative importance of these factors depends on age.

**Supplementary information:**

The online version of this article (10.3758/s13414-021-02242-z) contains supplementary material, which is available to authorized users.

## Introduction

In the visual environment, we are constantly changing the locus of our visual attention in order to focus on some locations or objects, while ignoring others. In the last decades, the topic of ‘scene viewing’ has received broad attention by eye-movement researchers, who have investigated what factors guide visual attention, and have accordingly implemented models of eye-movement control in scene perception. To date, studies have focused mainly on the effects of either image features (saliency; e.g., Foulsham & Underwood, 2008; Itti & Koch, 2000) or the meaning and structure of images (context; e.g., De Graef, Christiaens, & d’Ydewalle, 1990; Henderson, Weeks, & Hollingworth, 1999; Loftus & Mackworth, 1978), with substantial research investigating the relative contribution of these factors (e.g., Coco, Malcolm, & Keller, 2013; Spotorno, Tatler, & Faure, 2013; Underwood & Foulsham, 2006). Saliency and context are assumed to reflect, respectively, low- and high-level cognitive mechanisms guiding visual attention, and thus their study has been used to determine how much looking is a bottom-up, stimulus-driven process or a top-down process determined by higher-level factors. While detailed models of visual saliency have been proposed (e.g., Itti & Koch, 2000), it has become evident that these alone are not sufficient to explain gaze allocation in viewing (Tatler, Hayhoe, Land, & Ballard, 2011). Task demands, for example, can override the effects of low-level capture of attention (Foulsham & Underwood, 2007; Henderson, Brockmole, Castelhano, & Mack, 2007) and eye guidance can be driven by a process of locating “object entities” in scenes (object-driven models; Stoll, Thrun, Nuthmann, & Einhäuser, 2015), and, when searching for an object, preferentially looking at regions of the scene where the object is expected to be (Torralba, Oliva, Castelhano, & Henderson, 2006).

An often-neglected source of guidance in scenes – and one that rarely features in models of scene viewing – is that provided by the eyes of another. The pioneering eye-tracking works by Buswell (1935) and Yarbus (1967) showed that we fixate people more than the background of an image, with a strong preference to fixate faces and eyes when these are visible (see also Birmingham, Bischof, & Kingstone, 2008; Henderson, Falk, Minut, Dyer, & Mahadevan, 2000; Humphrey & Underwood, 2010). Moreover, not only do humans have a tendency to look at faces and eyes of others, but also to follow the gaze of another individual (Friesen & Kingstone, 1998), a tendency that develops from as young as 12 months (Thoermer & Sodian, 2001). Numerous studies show that this gaze-following behaviour facilitates target detection on a Posner-like (1980) gaze-cueing task where participants see a picture of a face with the eyes looking left or right, and then see a target (to which they have to respond) appearing in a position congruent or incongruent with the gaze direction. The main finding is that targets are detected more quickly when they appear at the congruent compared to the incongruent location (e.g., Friesen & Kingstone, 1998). However, this research into gaze following used mostly isolated faces as stimuli (e.g., Friesen & Kingstone, 1998; Ricciardelli, Bricolo, Aglioti, & Chelazzi, 2002). Only a few more recent studies employed a more naturalistic setting by presenting participants with real-world scenes. In particular, Kuhn and colleagues (Freebody & Kuhn, 2018; Kuhn, Vacaityte, D'Souza, Millett, & Cole, 2018) showed that observers fixate faster and for longer on objects in real-world scenes that are gazed at by an actor, compared to not looked at objects (see also Freeth, Chapman, Ropar, & Mitchell, 2010, and Riby, Hancock, Jones, & Hanley, 2013, who used scenes but tested clinical populations).

In the scene-viewing literature, only a handful of studies have looked at how perceived human gaze influences where our eyes fixate, and these studies employed relatively unconstrained viewing tasks. Castelhano, Wieth, and Henderson (2007), for example, asked participants to view a sequence of scenes portraying a story featuring an actor who, on the critical scenes, was looking at a particular object. Participants were asked to pay attention to the scenes, in order to understand the story, and each scene was presented for 5 s. Fixations to the object gazed at by the actor were compared to fixations to equivalent (matched in area and distance from the center) control regions. Results showed that gazed-at objects were looked at sooner and for longer compared to control objects. More recently, Borji, Parks, and Itti (2014; see also Langton, O’Donnell, Riby, & Ballantyne, 2006, and Recasens, Khosla, Vondrick, & Torralba, 2015, for related work) reported similar effects of gaze attracting visual attention when participants freely observed scenes, and further showed that gaze was a stronger cue than visual saliency. There is, thus, evidence that gaze influences scene viewing, but this evidence is almost restricted to tasks where participants observe real-world scenes in a relatively unconstrained manner (but see Kuhn et al., 2018, for a target-detection task in Experiment 2).

Understanding scene viewing requires not only that we can identify these different factors that are known to influence viewing behaviour, but also that we can quantify the relative importance of them. One of the main tasks that has been used to assess the relative importance of different factors in eye guidance during scene viewing is visual search, where participants are instructed to look for a previously named target object in an image (say, search for a cocktail in a barroom scene; Henderson et al., 1999). Studies of visual search in complex scenes have clearly demonstrated that the semantic context of a scene strongly affects visual guidance during object search. The representation of a complex visual scene includes semantic interpretation, alongside visual detail information, because a scene is a semantically coherent spatial arrangement of background elements and objects representing a real-world environment. That is, there is a meaningful context wherein objects are inserted, allowing us to understand rapidly what we are looking at (say, a kitchen). This is why research in scene perception has investigated extensively how the semantic context of a scene guides visual attention. We can perceive the meaning or gist of a scene within a glance of only 100 ms (Potter, 1976), and thereby activate expectations about the typical contents and spatial layout of such a ‘scenario’. This information may then help us decide where to move our eyes next, if we want to gather more precise information using our high-acuity foveal vision. Neider and Zelinsky (2003), for example, had participants looking for either a blimp or a jeep in scenes that had a low-lying desert region and an upper blue-sky region. Observers made more fixations to the sky region when searching for the blimp, and to the desert region when looking for the jeep, indicating their use of the scene’s semantic context to guide search. Indeed, context can guide eye movements even from the first moments after a scene appears, with the first eye movement already being directed toward regions of a scene in which the target is expected to be found (Spotorno, Malcolm, & Tatler, 2014; Torralba et al., 2006).

One way of investigating how scene context guides eye movements during search – and indeed during other visual tasks – is to test the effects of object-scene inconsistencies, and this method has become widely used since it was introduced as a way of testing semantic guidance in scenes by Loftus and Mackworth (1978). A consistent finding in the literature is that an object that is semantically inconsistent with a scene (e.g., a hairdryer in a kitchen) is fixated for longer than an expected object (e.g., a knife in a kitchen). One explanation for this is that inconsistent objects may involve an extra processing demand compared to consistent objects, whose features are already expected by the activated scene context. Evidence for this behaviour comes not only from studies of visual search (e.g., Cornelissen & Võ, 2017; De Graef et al., 1990), but also from studies using a range of tasks involving scene viewing such as recognition memory (e.g., Loftus & Mackworth, 1978; Võ & Henderson, 2009), free viewing (e.g., Bonitz & Gordon, 2008), or change detection (e.g., Coco, Nuthmann, & Dimigen, 2019; LaPointe, & Milliken, 2016; Underwood, Templeman, Lamming, & Foulsham, 2008). Concerning their potential to guide initial attention, objects that are inconsistent with the scene in which they are placed form a semantic outlier and, as such, they may be expected to attract visual attention early in scene viewing (Neider & Zelinsky, 2003; Spotorno et al., 2014; Torralba et al., 2006). However, evidence for this possibility is mixed and this remains a topic of continued debate in the literature, with some studies showing earlier fixations on inconsistent objects (e.g., Borges, Fernandes, & Coco, 2019), but others showing earlier fixations to consistent objects (e.g., Spotorno & Tatler, 2017; Võ & Henderson, 2011), or failing to find any modulation by semantic consistency of the time observers take to first fixate the target (Underwood & Foulsham, 2006; Võ & Henderson, 2009).

It is, thus, well established that visual search in scenes (and, more broadly, scene viewing) are strongly affected by the semantic or contextual information of the scene. Conversely, despite the recognition of its importance since the earliest work on eye movements in scene viewing, little research has investigated how scene viewing can be affected by gaze. Scene-perception studies showed that gazed-at locations in images attract visual attention (e.g., Castelhano et al., 2007), but it is not clear whether these effects, found during unconstrained viewing tasks, would also operate in a search task, especially given the finding that the relative importance of different potential sources of guidance in a scene is strongly influenced by the task viewers are performing. For example, viewing freely or for scene memorization may encourage attention to details and therefore be more affected by image properties such as visual saliency, whereas searching for a pre-specified target object may promote a top-down viewing strategy based on semantic context and the concurrent expectations for the target location (for discussion, see, e.g., Foulsham & Underwood, 2007; Spotorno & Tatler, 2017; Tatler et al., 2011; Underwood & Foulsham, 2006). Most of the evidence for strong effects of gaze cues on guiding visual attention comes from laboratory-based target-detection tasks with isolated faces, which have been criticised for their artificial nature and lack of social context (e.g., Risko, Laidlaw, Freeth, Foulsham, & Kingstone, 2012; Skarratt, Cole, & Kuhn, 2012). A question therefore arises concerning whether human gaze functions to guide visual search in complex scenes, where other cues such as the scene context are known to operate, and, if so, what are the relative contributions of gaze and contextual cues for the allocation of visual attention in such search tasks. Note that although, as mentioned above, there is evidence for a modulation of search performance for gazed-at targets in scenes (Kuhn et al., 2018, Experiment 2), the target to be detected in that task was a horizontal or vertical line superimposed to the scene and not a meaningful object of the scene. Here we are interested in the search for objects naturally belonging to the scene, which allows us not only to provide an even more naturalistic setting for the search task but also to investigate how the scene context containing the target object affects performance, alongside gaze cueing.

If we are to understand the relative contributions of context and gaze cues on visual search in complex scenes, it is important to acknowledge the fact that this is likely to vary over the course of adult ageing. Most of the literature that has contributed to our current understanding of context effects in scene search and the effects of eyes as cues for attentional allocation come from studies of young adults. However, studies have shown that both contextual and gaze-cueing effects differ in older adults. For example, contextual cueing in scene viewing seems to be of special relevance for older people. Neider and Kramer (2011; see also Borges et al., 2019) had younger and older adults looking for a blimp, a jeep, or an oleh (a ‘new’ object presented to participants) in pseudo-realistic scenes displaying the ground and the sky. The blimp and the jeep appeared in their ‘consistent’ locations (scene-constrained object), that is, on the sky and on the ground, respectively, and the oleh (scene-unconstrained object) appeared equally likely on the ground and on the sky. Younger and older participants were more accurate and faster at locating the scene-constrained blimp and jeep objects than the scene-unconstrained oleh object, but the differences in both accuracy and reaction times were greater for the older than for the younger group. This result is consistent with the suggestion that older adults rely more on contextual information to guide search (see Zanto & Gazzaley, 2014 for a review). This could be to compensate for an age-related impairment in cognitive functions such as attention and executive control that would underlie the worse performance displayed by older adults compared to younger adults on target search tasks (Lindenberger & Mayr, 2014; Madden, 2007; Watson, Maylor, & Bruce, 2005), or alternatively it might be to compensate for visual perceptual decline in healthy ageing (Monge & Madden, 2016).

In contrast, evidence suggests that older adults may use gaze cues less than younger counterparts. Using simplified paradigms such as the Posner-like gaze-cueing paradigm, Slessor, Phillips, and Bull (2008; see also Slessor, Laird, Phillips, Bull, & Filippou, 2010; Slessor et al., 2016) found that, in a typical gaze-cueing task, the cueing effect (faster response to targets at the gazed-at location than to targets at the non-gazed-at location) was significantly weaker for older than younger adults, and Kuhn, Pagano, Maani, and Bunce (2015) found that older participants made fewer anticipatory saccades (i.e., eye movements performed after the gaze cue but before the target presentation) towards the cued location than younger participants. Yet, no comparable evidence exists for scene viewing. The reasons for such age-differences in gaze following remain under debate, and competing explanations appeal to either general cognitive functioning decline such as impaired inhibition of irrelevant information or to a more specific social impairment in the ability to engage in joint attention, with general impairment of visual functions such as contrast sensitivity and even the time-course of effects also playing a role (Slessor et al., 2008; see Slessor et al., 2016, for discussion).

To sum up, little research to date has looked at how perceived gaze direction influences visual search in real-world scenes, where semantic context is a factor known to affect viewing behaviour. Moreover, it is not known how gaze and semantic cues might interact in guiding search, and whether the use of these cues might differ with ageing. In the present study, we tested the influence of semantic and gaze cues in visual search in scenes, and their relative importance for guiding search, in younger and older adults.

## The present study

To address these issues, we devised a visual search eye-tracking experiment where we manipulated whether the target object in a scene was gazed at or non-gazed at by a person, and whether it was semantically consistent or inconsistent with the gist of the scene. Our first question was whether perceived gaze direction influences visual search for objects in scenes, much as it facilitates search for non-object targets superimposed on scenes (Kuhn et al., 2018). Our second question related to how object-scene inconsistencies modulate visual search in scenes. We examined the effects of inconsistencies by manipulating the semantic relationship between the target object and the scene. Varying both gaze and consistency allowed us to assess the relative contribution of gaze and semantic cues for guiding eye movements during search. We tested older and younger participants, in order to determine whether there are age differences in the use of gaze and semantic cues in visual search.

Building on the findings reviewed above, we predicted that gazed-at objects and semantically consistent objects should lead to more efficient visual search. Further, the ageing literature suggests that older adults may be more influenced than younger subjects by semantic consistency, but less influenced by gaze cues. We examined the effects of gaze and consistency by analysing both the time participants took to answer whether the object was present in the scene and three eye-movement measures commonly reported in scene-perception studies: the time to first fixate the target, the probability of having fixated the target at the first few fixations, and the total time fixating the target. The first two of the eye-movement measures (time to first fixation and probability of having fixated the target in early fixations) capture the potential of an object to attract early attention and reflect object identification, whereas the last one (total fixation time at the target) captures later stages of attention and reflects object processing and recognition (e.g., Borges et al., 2019; Henderson et al., 1999; Underwood et al., 2008).

## Method

### Participants

We initially recruited forty-two older adults ranging in age from 60 to 81 years (M = 71.41, SD = 5.14), and forty-seven younger participants ranging in age from 19 to 24 years (M = 23.51, SD = 3.46). Older participants were recruited through the participant panel of the University of Aberdeen, who are usually invited to take part in psychology experiments, and the younger adults were recruited among students from the University of Aberdeen. Older participants were screened for mild cognitive impairment through the Montreal Cognitive Assessment questionnaire (Nasreddine, Phillips, Bédirian, et al., 2005), and all achieved a score greater than 23, the cutoff point recommended by Carson, Leach, and Murphy (2018). All participants had normal or corrected-to-normal vision, gave written informed consent, and were paid £10 for their participation. The study was approved by the Psychology Ethics Committee from the University of Aberdeen.

From the initially recruited 89 participants, we could not fully record the eye movements of 22 participants as we could not obtain a valid initial calibration of the eye-tracker, mainly because of the use of strong eyeglasses or varifocal lenses that caused distortions. We also did not accept the data from a participant who answered correctly on only 30% of trials (all other participants answered correctly on more than 80% of trials). All analyses below are therefore reported for 30 older (11 males; age M = 70.8 years, SD = 5.7) and 34 younger (three males; age M = 23.0 years, SD = 2.9) participants.

### Design and materials

**Fig. 1:**
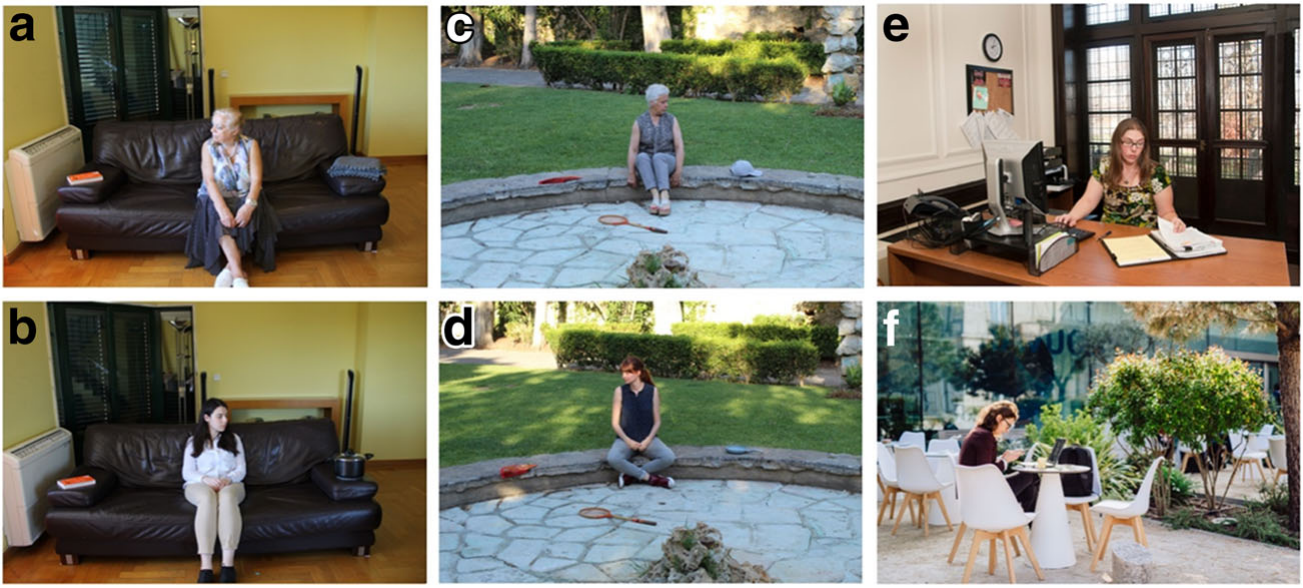
Representative sample of the photographs used as stimuli. (**A**) and (**B**) are images of an indoor scenario (living room) for the consistent (throw) non-gazed condition (**A**), and the inconsistent (pot) gazed condition (**B**). (**C**) and (**D**) are photos of an outdoor scenario (garden) for the consistent (cap) gazed condition (**C**), and the inconsistent (frying pan) non-gazed condition (**D**). (**E**) and (**F**) are examples of two filler images

The experimental items for each participant were 32 real-world scenes (i.e., photographs) where participants had to look for a pre-specified (named) target object, while their eye movements were recorded. In each scene (e.g., living room), an actor was looking at the to-be-searched target object (e.g., throw) or looking at a distractor object on the opposite side of the scene, and the target object could be consistent (e.g., throw) or inconsistent (e.g., pot) with the semantic context of the scene (e.g., living room in Fig. 1A and B). Sixty-four additional photographs were filler items, obtained freely from web sites, that appeared between each two experimental items. Filler items were intended to make the experimental manipulation not transparent to participants (e.g., noticing that the person in the scene was always gazing at either the target or the object in the opposite direction), and thus avoid the development of any possible search strategies. Moreover, whereas in the 32 experimental items the target object was always present, for 48 of the fillers the object to be searched for was absent. For each trial the target item was cued by a word appearing on the screen before the presentation of the image, then participants had to identify whether the target object was present in the scene. In half of the 96 trials the correct answer to this question was Yes, and it was No for the other half of presented items.

To create our 32 experimental items, we took coloured photos with a Canon EOS 4000D (miniature versions of the 256 photos used are found in the Online Supplementary Materials). We photographed 32 different scenes (16 outdoor, e.g., garden, and 16 indoor, e.g., living room). For each of the (16) indoor scenes, four young (one male) and four older (two males) actors were photographed, and for each of the (16) outdoor scenes another four young (two males) and four older (three males) actors were photographed. Having younger and older actors in the scenes was intended to avoid any potential confound of an own-age bias (e.g., Freebody & Kuhn, 2018; Slessor et al., 2010). Actors were recruited through word of mouth, gave written informed consent for the use of the photographs to the study, and were paid £15 for their time. For each item (e.g., living room) and for each actor we first placed the consistent object (e.g., throw) in the physical scene, and the distractor object (which was always consistent with the scene) at the opposite side, relative to the actor. We photographed the actor looking at the target object, first, and then looking at the distractor. This procedure was then repeated, after replacing the consistent by the inconsistent (e.g., pot) target object. Photographs were taken with a tripod that was kept at the same location and with the same extension for all photographs of each scene. Each photo was saved as a JPG file with 5,184 × 3,456 pixels dimension, and 72 dpi horizontal and vertical resolution, and was then resized to 1,620 × 1,080 to fit the presentation screen (see Procedure) while keeping the same aspect ratio. There was no photo-editing of our stimuli.

We had 256 unique items corresponding to eight versions of each scene containing a target object (located at the right (15) or left (17) halves of the photograph) that was either semantically consistent or inconsistent with the scene (C, I), gazed at or not gazed at (G, NG) by the actor in the picture, and where the actor was young or old.^1^ We created eight lists of stimuli, with one version of each scene appearing on each list (Latin square design). Each list also contained the 64 fillers, with the total number of trials being 96. The order of presentation of critical items and fillers was randomized, with the constraint that at least one filler was presented between every two experimental items. Young and older (Y, O) participants each completed one of the eight lists, so that, for each scene (item), we obtained an equal number of observations for each condition crossing gaze and consistency (and age of the actor).

The design of interest for our analyses was a 2 (consistency, C vs. I) × 2 (gaze, G vs. NG) within-participants design, with Younger and Older (group, Y vs O) participants being a between-participants factor.

### Apparatus and recording

The experiment was generated in SR Research Experiment Builder 1.10.165 (2011), and conducted on an Asus TX650 computer running OS Windows7 Pro. Scenes were presented on a BenQ XL2420Z 24-in. monitor with 1,920 × 1,080-pixel image resolution, and a refresh rate of 120 Hz. Eye movements were recorded using an EyeLink 1000 desk-mounted eye-tracker at a sampling rate of 1,000 Hz. Participants sat 72 cm away from the display, and a forehead and chin rest was used for head stabilization. Viewing was binocular but only the participant’s dominant eye was tracked, as determined by a parallax test. The experiment began with a 9-point calibration and validation procedure. Calibration was accepted if the average and worst calibration errors were below 0.5° and 1° of visual angle, respectively. A new calibration was repeated whenever the experimenter found it necessary – namely, when the pre-trial calibration check shown at each trial onset indicated an error above 1° for three or more successive trials.

### Procedure

After the initial calibration procedure, participants were presented with a written instruction of the task, saying they should search for pre-specified objects in the scenes. If the target object was present, participants should look at it and respond Yes, and if it was not present, they should simply answer No (with the L and S keys, respectively, marked as YES and NO on the keyboard).

**Fig. 2:**
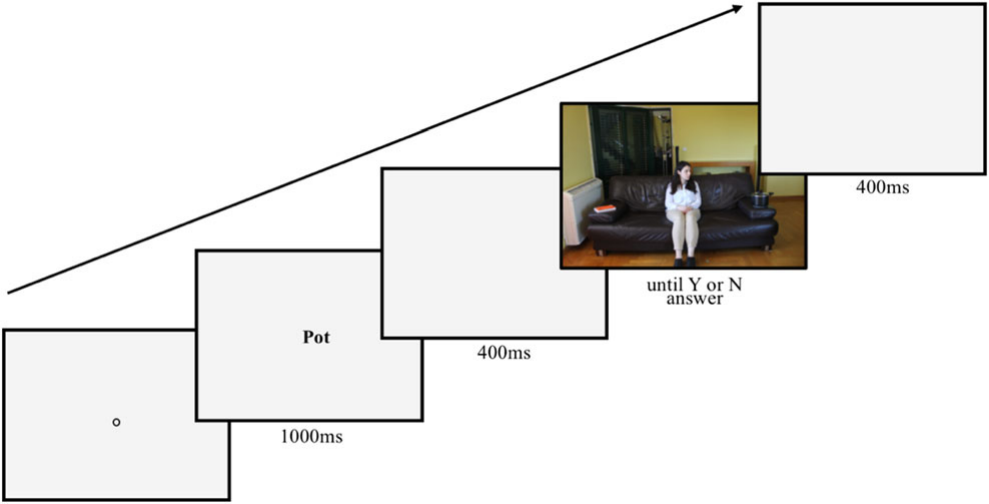
Example trial of the visual search experiment. Participants would first fixate a central dot for a pre-trial calibration check, and then see a written word indicating which object to search for, followed by a blank screen and the search scene containing (or not) the target object. Participants were instructed to look at the target object if it was present, and to press the key on the keyboard to log their yes/no response, and, after a blank screen, the next trial would start

A schematic representation of a trial is shown in Fig. 2. Each trial started with a pre-trial calibration check. If the error was <1°, the experimenter would accept the fixation, triggering the presentation of a blank screen for 400 ms. Then, the name of the target object was presented for 1,000 ms, after which a blank screen was presented for 400 ms. Afterwards, the search scene was presented, remaining on the screen until a Yes or No answer was given by the participant. After pressing the answer key, a 400-ms blank screen finished the trial, and the next trial began. A block of practice consisting of eight additional trials was administered before the experiment began.

### Analysis

The region of interest (ROI) for the eye-movement analysis was the target object. We defined ROIs using MATLAB’s (version R2019a) function *drawpolygon* to draw, in each scene, the contour of the target object. For each scene, each actor was photographed looking at or away from two different target objects (consistent and inconsistent). To ensure that there were no significant differences between low-level visual properties of consistent and inconsistent target objects, we computed the salience of each of the 256 scenes using the Adaptive Whitening Saliency model (Garcia-Diaz, Fdez-Vidal, Pardo, & Dosil, 2012). We first normalized the saliency map so that values of each pixel ranged between zero and one, and then calculated the mean of the values that belonged to the target ROI. Paired (for each scene) *t* tests on the mean salience of the target region showed that salience did not differ significantly for consistent (M = 0.29, SD = 0.16) and inconsistent (M = 0.29, SD = 0.15) objects (t (31) = -0.11, p = 0.91). The consistent and inconsistent target objects likewise did not differ significantly on their size (t (31) = .89, p = 0.07).

Raw gaze data were pre-processed using MATLAB. For each participant and trial, we first extracted the timestamps indicating the trial start and end, the scene onset, the manual response, and the fixations (start, end, *x* coordinate, and *y* coordinate) starting between the scene onset and the final blank screen. We then mapped each fixation onto the target ROI. We assigned a fixation to the ROI if its distance to the nearest ROI pixel was smaller than the distance corresponding to 1° of visual angle (47.3 px). From the 2,048 trials (32 experimental trials for each of the 64 participants), we removed 17 trials for which the average and worst calibration errors were below 0.5° or 1° of visual angle, respectively. We further discarded from analyses 128 trials where participants answered incorrectly that the object to be searched for was not present. We also removed from the analysis the trials where the target was not fixated at all (36) or where, at the scene onset, the target region was already being fixated (16). These procedures led to elimination of 197 out of 2,048 trials (9.6%).

We focused on measures that are commonly reported in studies on scene viewing and visual search: (a) the time to first fixate the target (i.e., the start time of the first fixation at the target minus the scene onset time, in ms); (b) the probability of having fixated the target at each fixation ordinal number (i.e., whether, at each fixation ordinal number, the target was or was not fixated; a binomial coded 1 and 0, respectively); (c) the total fixation time at the target (i.e., the sum of the durations of all fixations at the target starting between the scene onset and the final blank screen, in ms); and (d) the answer response time (i.e., the difference between the manual response time and the scene onset, in ms). In order to obtain closer to normal distributions of the time measures (a, c and d), we log-transformed these outcomes. The choice of the log transformation was based on the estimation of the optimal values of the λ-coefficient for the Box-Cox power transformation, which were -0.14, 0.19, and -0.50, for each of the measures. These are closer to 0 (the estimate for which a log transformation is used) than to 1 or -1 (used for keeping the original metric or using the reciprocal transformation, respectively), making log transformation more appropriate for our data (Box & Cox, 1964; Venables & Ripley, 2002; as cited by Kliegl, Masson, & Richter, 2010). In addition, we assessed data normality through visual inspection of Q-Q plots and lines. We further removed latencies that distanced more than three standard deviations (SDs) from the mean, for each participant. This corresponded to six (0.3%) and 13 (0.7%) trials, for time to first fixate and total fixation time, respectively. For the answer response time (RT), we first excluded 17 outlier observations longer than 5,000 ms (0.9%) and then, for the log-transformed RTs, another three observations (0.1%) more than 3 SDs away from the mean.

We fitted to our outcomes linear-mixed effects models (LMM; Baayen, Davidson, & Bates, 2008), as implemented by *blme* (vs. 1.0-4; Dorie, 2015) and *lme4* (Bates, Mächler, Bolker, & Walker, 2015) packages in *R*. LMMs avoid data aggregation (e.g., across items), a practice that may lead to less precise estimates, especially with small samples (Muth et al., 2016); they allow a simultaneous estimation of between-participants and between-items (i.e., scenes) variance; and they are robust in dealing with data loss and not fully balanced designs (Kliegl et al., 2010). We had participants (64) and items (32) as random factors, and our fixed effects (all contrast coded by centring) were: Group (between-participants; coded: O, -0.54; Y, 0.46), Consistency (within-participants; coded: C, -0.5; I, 0.5), and Gaze (within-participants; G, -0.48; NG, 0.52^2^). We fitted full models (all main effects and possible interactions) with a maximal-random structure when justified by the design (Barr, Levy, Scheepers, & Tily, 2013). In particular, there was no random slope for Group (as participants belong to one group only), and we only introduced random slopes for fixed effects that allowed the model to converge (see the notes in the Tables for the models’ syntax). We report the predictors’ coefficients (β values), SE, *t* values, and the derived *p* significance values (by treating the t-statistic using the standard normal distribution as a reference; e.g., Baayen et al., 2008, footnote 1).

## Results

On each scene, the target object could be gazed at or non-gazed at by the photographed actor, and it could be semantically consistent or inconsistent with the meaning of the scene. Our results thus reflect how gaze and consistency (fully crossed within-participants categorical predictors) influenced visual search by younger and older participants (between-participants categorical predictor). We first present the results on the measures reflecting early capture of visual attention, i.e., time to first fixate the target and probability of having fixated the target at the initial fixations, and then show results on measures capturing later stages of processing in the task (total time fixating the target and reaction time to answer).

### Time to first fixate the target

**Fig. 3:**
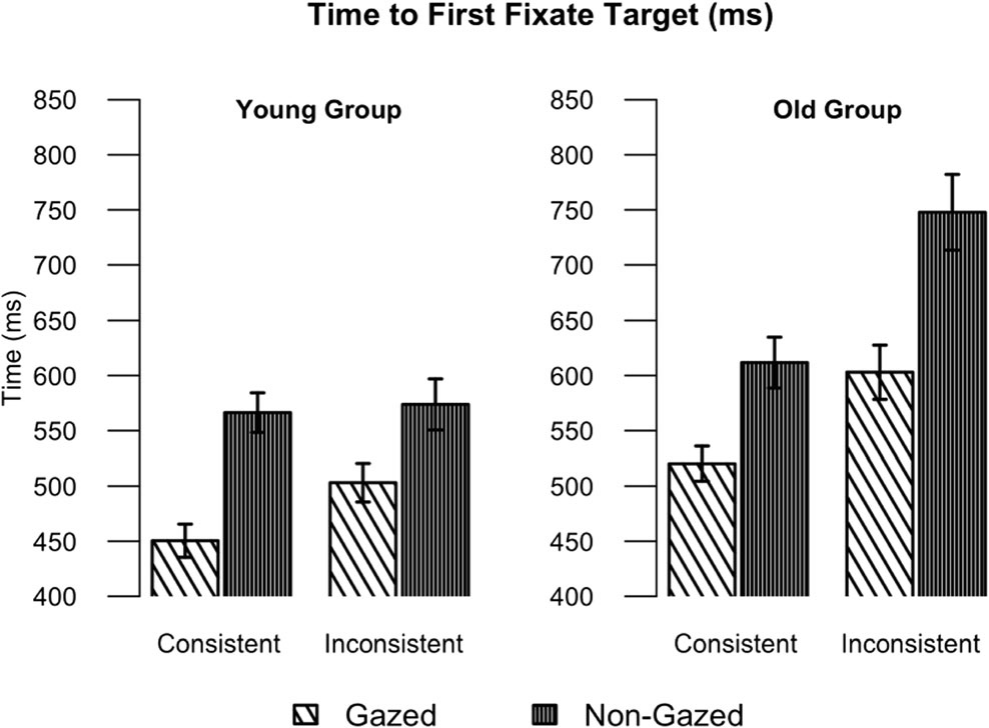
Mean time to first fixate the target for the younger (**left**) and older (**right**) participants, showing the effects of gaze and object consistency. Error bars represent standard errors on means

The time elapsed between the onset of the search scene and the first fixation at the target object is a measure that reflects the capacity of the object to attract early visual attention. The analysis of this measure can thus inform about initial effects of gaze and semantic consistency on the eye movements during search. Figure 3 shows the effects of gaze cue, consistency and age group on the time to first fixate the target, and Table 1 presents the summary of the fitted model to log-transformed times. (Here and in all other figures in the Results section, we present the means of the non-transformed measures, for easier visualization and interpretation.)

**Table 1:** Summary of the linear mixed effects model (LMM) fitted to the log-transformed time to first fixate the target.

Time (log(ms)) to First Fixate Target				
Predictors	Estimate	SE	t value	p value
(Intercept)	6.22	0.05	137.27	<0.01
group [O, -0.53; Y, 0.47]	-0.16	0.04	-3.69	<0.01
gaze [G, -0.49; NG, 0.51]	0.17	0.03	6.66	<0.01
consistency [C, -0.5; I, 0.5]	0.10	0.02	5.24	<0.01
group:gaze	0.01	0.04	0.33	0.74
group:consistency	-0.07	0.04	-1.74	0.08
gaze:consistency	-0.02	0.04	-0.59	0.55
group:gaze:consistency	-0.17	0.07	-2.35	0.02

Note: the syntax of the model is blmer(Time ~ 1 + group + gaze + consistency + group:gaze + group:consistency + gaze:consistency + group:gaze:consistency + (1 | item) + (1 | subj) + (0 + group | item) + (0 + consistency | subj) + (0 + gaze | subj) + (0 + gaze | item), data = dataset, control = lmerControl(optimizer = "Nelder_Mead"))

Older participants displayed overall longer times to first fixate the target (main effect of group). Across both groups, participants fixated the target object sooner when it was gazed at by the actor in the scene, compared to when it was not gazed at. This main effect of gaze (~86 ms) is evidence of an influence of perceived gaze direction on visual search for objects in real scenes.

In addition, for both groups, participants were faster (~50 ms) to fixate target objects that were semantically consistent with the scene, compared to semantically inconsistent objects. However, the effects of consistency and gaze were qualified by a significant three-way interaction between group, gaze and consistency. To explore further this interaction, we conducted follow-up LMMs where, for each group (younger and older participants), we created a four-level variable combining the two consistency levels with the two gaze levels. This variable was contrast coded so that we had three contrasts: Gazed-Consistent versus Gazed-Inconsistent, NonGazed-Consistent versus NonGazed-Inconsistent, and Gazed versus NonGazed (for each contrast, -0.5 vs. 0.5). Earlier fixation of the target was observed for gazed-at objects, compared to non-gazed-at ones, for both groups (Gazed vs. NonGazed contrasts; younger: β = 0.18, SD = 0.03, p<.01; older: β = 0.16, SD = 0.04, p<.01), indicating that gaze benefited search for younger and older adults. Older adults fixated sooner consistent target objects relative to inconsistent targets, in both the gazed-at (β = 0.10, SD = 0.05, p = .03) and non-gazed-at (β = 0.17, SD = 0.04, p<.01) conditions, suggesting that they used the semantic consistency cues regardless of gaze cues. However, younger adults fixated consistent targets sooner than inconsistent targets when these were gazed at (β = 0.12, SD = 0.04, p<.01), but when the target objects were not gazed at the contrast of consistency was not significant (β = 0.01, SD = 0.04, p = .72).

A possible explanation for why consistency would not affect the time younger participants took to first fixate targets that were not gazed at is that, in this case, the distractor object instead was looked at by the actor, and visual attention could be attracted to it to an extent that would override the effects of the consistency cues. To explore this possibility, in the next section we carried out an exploratory analysis of the probabilities of fixating both the target and the distractor objects at the initial and subsequent recorded fixations.

### Probability of fixating the target and the distractor after scene presentation

**Fig. 4:**
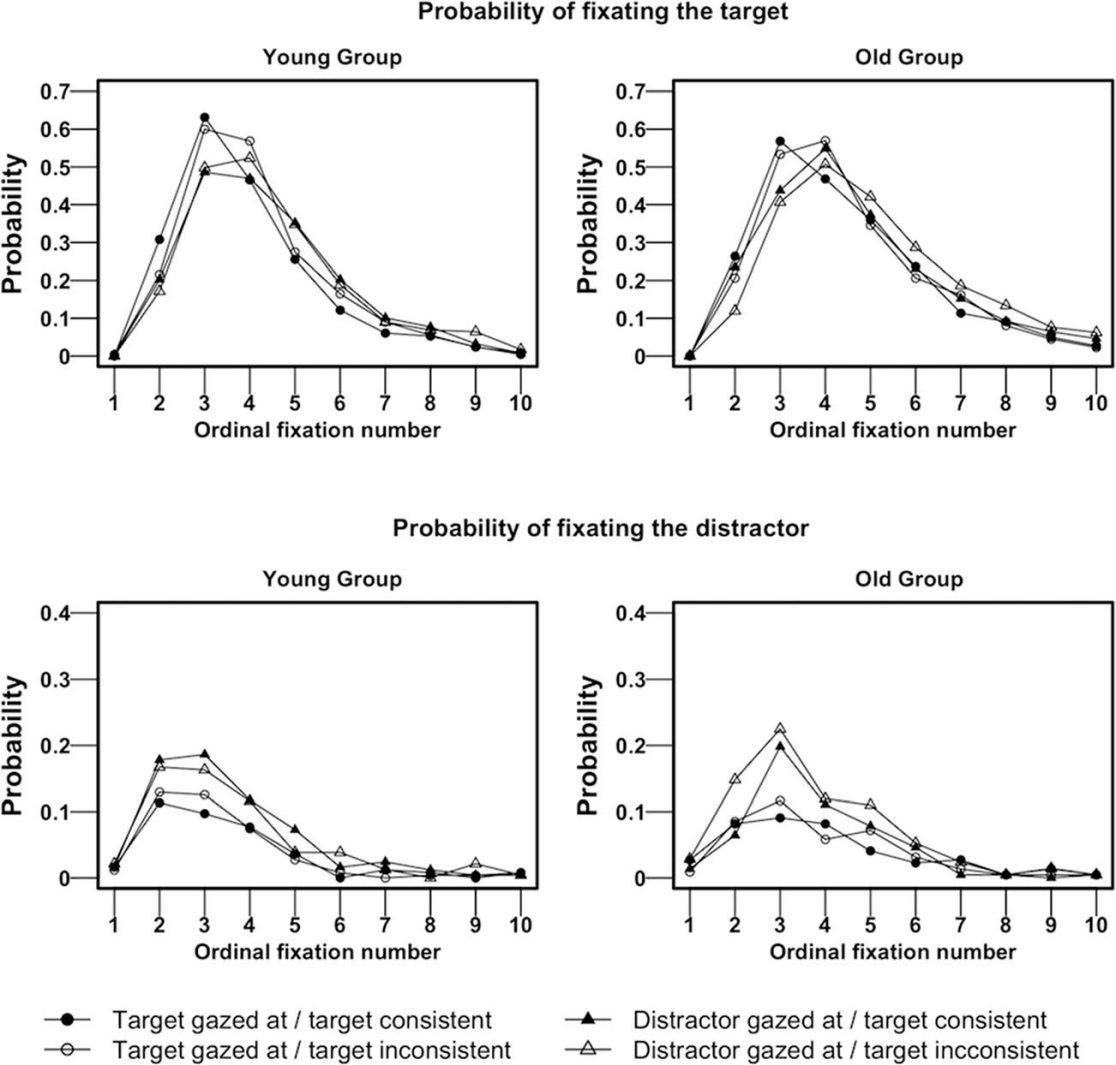
Mean probability of participants’ fixation to the target (**top row**) and to the distractor (**bottom row**), at each ordinal fixation number, when the target was gazed at by the actor (circles) and when the distractor instead was gazed at by the actor (i.e., the distractor-gazed-at condition; triangles). Solid points represent the semantically consistent target condition, and non-filled points represent the semantically inconsistent target condition. The data from the young and older groups are visualized in the left and right panels, respectively

While time to first fixate a target offers a measure of how soon that object is selected for foveal scrutiny, it may miss subtle differences in how the target is searched for. Older adults might take longer to fixate the target because they tend to make more fixations and to re-fixate previously inspected areas during search (Maltz & Shinar, 1999; Veiel, Storandt, & Abrams, 2006). One alternative way to assess how target objects attract initial attention is to consider the probability that the target is fixated on each ordinal fixation during viewing. To do so, we computed the probabilities of fixating the target for each ordinal fixation number (from the first to the tenth fixations^3^) as the proportion of trials on which the target was fixated at that ordinal fixation number. We further considered the probability of fixating the distractor at the initial and subsequent recorded fixations, as the distractor object can compete with the target for initial attention, especially when it is gazed at by the actor (i.e., in the condition when the target is not gazed at). Figure 4 plots the probabilities of fixating the target (top row) and the distractor (bottom row), at each ordinal fixation, showing the effects of gaze and object consistency. Note that when the target was not gazed at by the actor in the scene, the actor was instead looking at the distractor. For easier interpretation, we thus named this condition ‘distractor gazed at’ (represented by the triangle points in Fig. 4).

The first fixation is the one that was ongoing when the scene appeared, not reflecting any active selection of information in the scene, and therefore it is not included in our analysis (see footnote 3). At the second fixation (resulting from the first eye movement performed after the scene presentation), the target was fixated in 22% of trials, and at the third fixation the target was fixated in 52% of trials (across conditions). We analysed the subsets of the data corresponding to the second and third fixations (e.g., Henderson et al., 1999). In each case we coded, for each trial, whether the target was fixated or not (1 vs. 0) at that fixation number, and we fitted to the binomial outcome a mixed logit regression (cf. Jaeger, 2008) using the *glmer* with a ‘logit’ link function in *R*. Our fixed effects (centred) were Group (O, -0.53; Y, 0.47), Consistency (C, -0.5; I, 0.5), and Gaze (G, -0.49; NG, 0.51). We proceeded in the same way and ran similar logistic regressions to assess the probability of fixating the distractor.

The probability of the participant fixating the *target* (Fig. 4, top row) at fixation number 2 was higher when the actor was looking at the target compared to looking at the distractor, and for consistent compared to inconsistent target objects (main effects of gaze and consistency, see Table 2a). The three-way interaction between group, gaze, and consistency was not significant. When analysing the probability of fixating the *distractor* (Fig. 4, bottom row) at fixation number 2 (Table 2b), we found that participants were more likely to look at the distractor when the actor was looking at it (i.e., when the target was not gazed at) as compared to the conditions where the actor was looking at the target (main effect of gaze). This indicates that participants followed gaze, even when it was focused in the direction of the distractor. Younger participants also fixated the distractor object more compared to older adults (main effect of group). Importantly, there was a significant three-way interaction between group, gaze and consistency. We ran follow-up models for each group, setting the contrasts as before (Gazed-Consistent vs. Gazed-Inconsistent, NonGazed-Consistent vs. NonGazed-Inconsistent, and Gazed vs. NonGazed; for each contrast, -0.5 vs. 0.5). We found that younger adults fixated the distractor significantly more when this object was looked at compared to trials when it was not looked at (Gazed vs. NonGazed contrast, β = 0.56, SE = 0.21, p = .01), and that consistency did not influence the probability of fixating the distractor (none of the two other contrasts was significant, ps > 0.2). On the contrary, the actor’s gaze direction was not a main predictor of how likely older adults were to initially saccade to the distractor (non-significant Gazed vs. NonGazed contrast; β = 0.24, SE = 0.28, p = .38). However, when the distractor was gazed at, in trials where the target was semantically inconsistent with the context, older adults were more likely to saccade to the distractor, compared to trials where the target was semantically consistent (NonGazed-Consistent vs. NonGazed-Inconsistent contrast, β = 1.15, SE = 0.28, p = .01). This suggests that older adults used both gaze cues (following it towards the distractor) and also semantic cues (fixating more likely the distractor if the target was inconsistent) to guide initial eye movements. The contrast between consistent and inconsistent target objects was not significant in the condition where the target was gazed at (β = 0.02, SE = 0.39, p = .97).

**Table 2:** Summary of the linear mixed effects model (LMM) fitted to the probability of fixating the target (a) and (b) the distractor at the second fixation

Predictors	a. Probability of fixating target second fixation				b. Probability of fixating distractor second fixation			
	β	SE	z value	p value	β	SE	z value	p value
(Intercept)	-1.70	0.23	-7.45	<0.001	-3.02	0.34	-8.77	<0.001
group [O, -0.53; Y, 0.47]	0.16	0.18	0.88	0.38	0.70	0.25	2.81	0.01
gaze [G, -0.49; NG, 0.51]	-0.54	0.13	-4.27	<0.001	0.41	0.16	2.50	0.01
consistency [C, -0.5; I, 0.5]	-0.58	0.13	-4.53	<0.001	0.30	0.17	1.83	0.07
group:gaze	-0.06	0.25	-0.22	0.83	0.34	0.33	1.04	0.30
group:consistency	0.17	0.25	0.65	0.51	-0.51	0.33	-1.54	0.12
gaze:consistency	-0.19	0.25	-0.76	0.45	0.25	0.33	0.75	0.45
group:gaze:consistency	0.92	0.51	1.80	0.07	-1.60	0.67	-2.39	0.02

Note: the syntax of the model is glmer(binomial measure ~ 1 + group + gaze + consistency + group:gaze + group:consistency + gaze:consistency + group:gaze:consistency + (1 | item) + (1 | subj), family = binomial(link = "logit"), data = dataset, control = glmerControl(optimizer = "bobyqa"))

The probability of fixating the target (Fig. 4, top row) at fixation number 3 was only affected by the actor’s gaze, with gazed-at targets fixated upon more than non-gazed-at ones, and by group, with younger adults fixating more to the target than older adults (Table 3a). At fixation number 3, looks to the distractor (Fig. 4, bottom row) were also a function of the actor’s gaze, with more fixations when the distractor was looked at, compared to when the target instead was gazed at, and no other effects were significant (Table 3b).

**Table 3:** Summary of the linear mixed effects model (LMM) fitted to the probability of fixating the target (a) and (b) the distractor at the third fixation

Predictors	a. Probability of fixating target third fixation				b. Probability of fixating distractor third fixation			
	β	SE	z value	p value	β	SE	z value	p value
(Intercept)	0.09	0.15	0.58	0.56	-2.15	0.21	-10.11	< .01
group [O, -0.53; Y, 0.47]	0.35	0.15	2.33	0.02	-0.11	0.15	-0.72	0.469
gaze [G, -0.49; NG, 0.51]	-0.63	0.10	-6.14	0.00	0.78	0.14	5.47	< .01
consistency [C, -0.5; I, 0.5]	-0.12	0.10	-1.17	0.24	0.16	0.14	1.15	0.25
group:gaze	-0.01	0.20	-0.03	0.98	-0.35	0.28	-1.25	0.211
group:consistency	0.04	0.20	0.19	0.85	-0.11	0.28	-0.38	0.702
gaze:consistency	0.12	0.20	0.59	0.55	-0.33	0.28	-1.17	0.244
group:gaze:consistency	0.18	0.41	0.45	0.66	-0.36	0.57	-0.64	0.521

Note: the syntax of the model is glmer(binomial measure ~ 1 + group + gaze + consistency + group:gaze + group:consistency + gaze:consistency + group:gaze:consistency + (1 | item) + (1 | subj), family = binomial(link = "logit"), data = dataset, control = glmerControl(optimizer = "bobyqa"))

### Total fixation time on the target

**Fig. 5:**
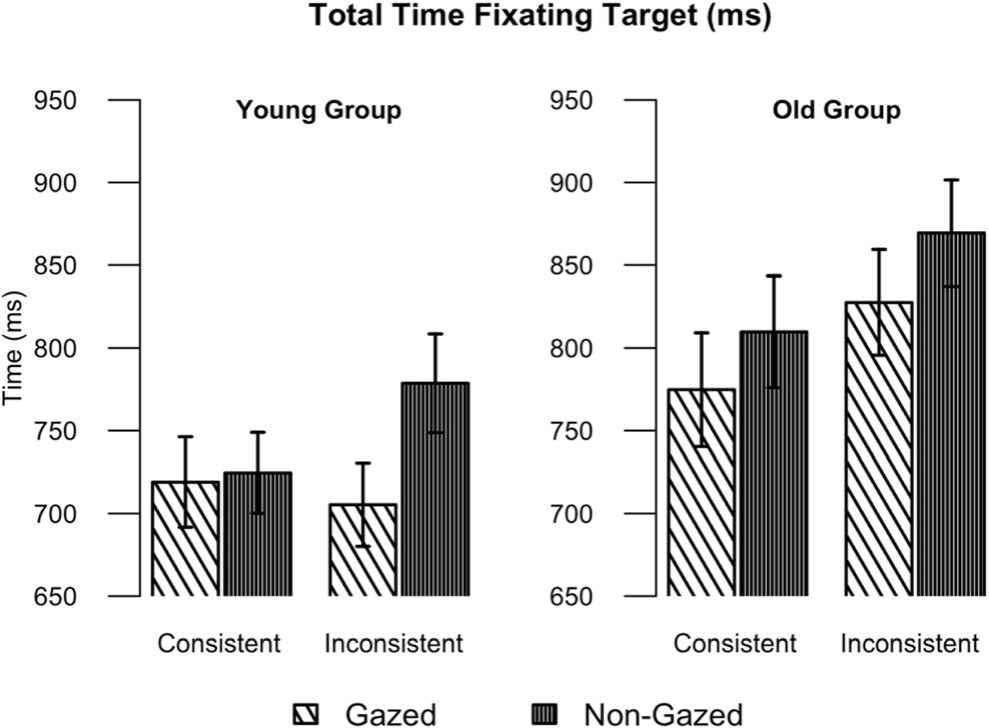
Mean total time fixating the target for the Younger (**left**) and Older (**right**) groups, in the conditions crossing Consistency of the object and Gaze. Error bars represent standard errors on means

Figure 5 presents the mean total time that participants spent looking at the target object, and Table 4 presents the fitted LMM model to (log-transformed; see Analysis) observations for total times. The total time participants spend fixating a target object is a commonly used measure to index the processing effort needed for correct recognition of the visual object and then deciding whether that object is the target.

**Table 4:** Summary of the linear mixed effects model (LMM) fitted to the log-transformed total time fixating the target

Total time (log(ms)) fixating target				
Predictors	β	SE	t value	p value
(Intercept)	6.49	0.05	125.03	<0.01
group [O, -0.53; Y, 0.47]	-0.11	0.09	-1.23	0.22
gaze [G, -0.49; NG, 0.51]	0.06	0.02	2.79	0.01
consistency [C, -0.5; I, 0.5]	0.05	0.02	2.45	0.01
group:gaze	0.00	0.04	0.01	0.99
group:consistency	-0.08	0.04	-1.88	0.06
gaze:consistency	0.02	0.04	0.42	0.67
group:gaze:consistency	0.05	0.08	0.57	0.57

Note: the syntax of the model is blmer(Time ~ 1 + group + gaze + consistency + group:gaze + group:consistency + gaze:consistency + group:gaze:consistency + (1 | item) + (1 | subj) + (0 + group | item) + (0 + consistency | subj) + (0 + gaze | subj) + (0 + gaze | item), data = dataset, control = lmerControl(optimizer = "Nelder_Mead"))

Again, we looked at the effect of age group, consistency and actor gaze. We found a main effect of consistency, whereby participants spent more time looking at inconsistent than consistent objects. We also found a main effect of gaze, indicating that participants of both groups fixated for longer at non-gazed-at target objects than gazed-at target objects. Although, as illustrated in Fig. 5, raw times were longer for older than for younger adults, our analysis on the log-transformed measures indicated that this age difference was not significant. Finally, there was a trend for older participants to spend more time looking at inconsistent objects, compared to younger participants and consistent objects, but the interaction between group and consistency did not reach significance.

### Manual response time

**Fig. 6:**
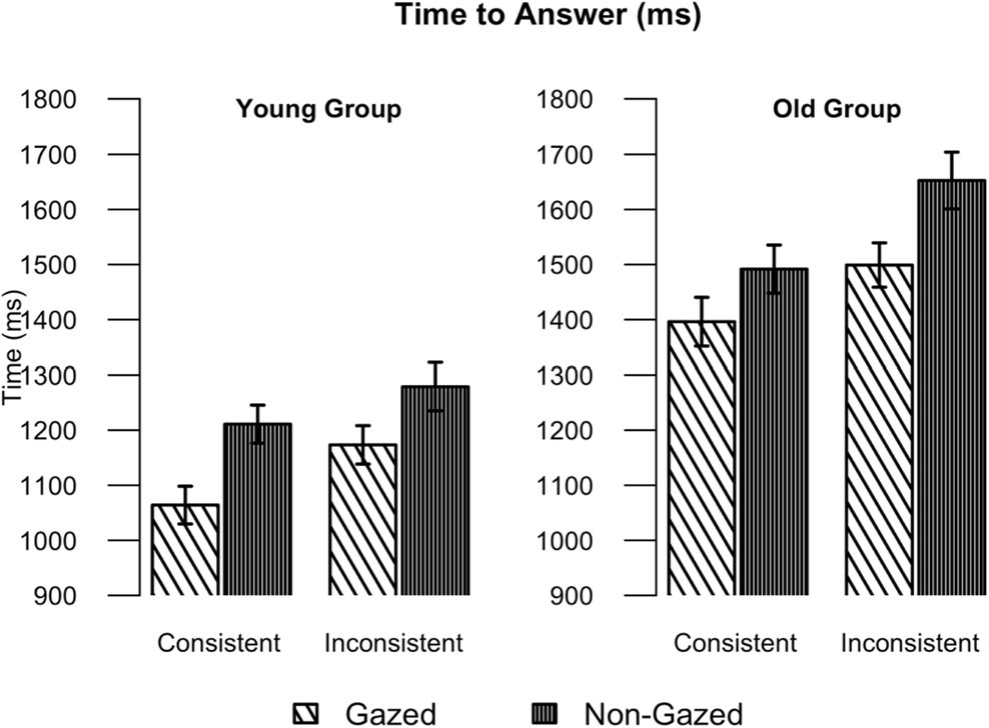
Mean time (ms) to answer for the Younger (**left**) and Older (**right**) groups, in the conditions crossing Consistency of the object and Gaze. Error bars represent standard errors on means

Finally, we analysed the time participants took to press the key indicating the target object was present in the scene. Figure 6 plots the mean response time (RT) for participants to answer that the target object was present in the scene, and Table 5 presents the summary of the fitted model to the log-transformed RT measures. We found that participants were faster to respond when the target object was semantically consistent with the scene compared to when it was semantically inconsistent (main effect of consistency, refer to Table 5). Participants were also faster to respond when the target object was gazed at, compared to when the object was non-gazed at (main effect of gaze). However, there was no significant interaction between gaze and group, nor between consistency and group, suggesting that age differences are not evident at this later stage of processing. The older group was overall slower than the younger one, as indicated by the main effect of group.

**Table 5:** Summary of the linear mixed effects model (LMM) fitted to the log-transformed time to answer

Time to answer (log(ms))				
Predictors	β	SE	t value	p value
(Intercept)	7.11	0.04	179.12	<0.01
group [O, -0.53; Y, 0.47]	-0.26	0.06	-4.46	<0.01
gaze [G, -0.49; NG, 0.51]	0.10	0.02	5.38	<0.01
consistency [C, -0.5; I, 0.5]	0.07	0.01	4.83	<0.01
group:gaze	0.03	0.03	0.90	0.37
group:consistency	-0.02	0.03	-0.55	0.58
gaze:consistency	-0.04	0.03	-1.46	0.14
group:gaze:consistency	-0.06	0.06	-1.10	0.27

Note: the syntax of the model is blmer(Time ~ 1 + group + gaze + consistency + group:gaze + group:consistency + gaze:consistency + group:gaze:consistency + (1 | item) + (1 | subj) + (0 + group | item) + (0 + consistency | subj) + (0 + gaze | subj) + (0 + gaze | item), data = dataset, control = lmerControl(optimizer = "Nelder_Mead"))

## Discussion

We investigated how gaze and semantic consistency influenced visual search in real-world scenes, with younger and older adults. We analysed both measures of the initial capture of attention, reflecting extrafoveal selection of target objects, and measures of later processing, reflecting foveal inspection of targets after overt attentional selection. We found that both gaze and consistency influenced search but, crucially, the two high-level sources of information interacted differently for younger and older participants in the early stage of search. In the older group, the mean time to first fixate the target was shorter for consistent compared to inconsistent targets, and for gazed at compared to ignored targets. However, for younger participants, the advantage of consistent target objects was only observed when those targets were gazed at. Such age differences were not evident in the time spent fixating the target or the overall manual response time, where we only found overall effects of gaze and consistency.

Our results extend current understanding of scene viewing in two ways. First, they show that gaze is an important factor influencing visual search in complex scenes, and should thus be incorporated in models of visual search and, more broadly, of scene viewing. Second, they highlight the importance of understanding the relative contribution of different factors to viewing behaviour and its potential variations across the lifespan.

Whereas some prior scene-viewing studies used stimuli with people and showed that locations or objects that were gazed-at attracted more fixations (Borji et al., 2014; Castelhano et al., 2007; Langton et al., 2006), in those studies the participants’ task was to either look relatively freely at each scene (i.e., free viewing, or general understanding), or to detect changes occurring in subsequent presentations of a scene. In contrast, in our search task, participants searched for a pre-specified target object, and thus they had prior knowledge of target features that they could integrate with the information from the scene context to guide visual attention in a top-down process. During search, visual guidance from the target template and from scene context may overshadow or alter the effects of gaze observed in free-viewing tasks. We asked whether similar effects of gaze in free-viewing and change detection would be observed in visual search, and hypothesised that, when viewing real-world scenes while searching for objects, people would perform better at finding targets that were gazed at by a person, compared to targets that were not gazed at. As predicted, the gaze direction of the actor in each photographed scene guided search for the target object early in viewing: Both young and older adults were faster to fixate on targets that were looked at by the actor in the scene than to fixate on ignored targets.

Many models of attention have focused on how viewing is modulated by bottom-up factors such as image salience and top-down information such as semantic context or task demands (see, for reviews, Findlay, 2003; Henderson, 2003; Tatler et al., 2011), but most did not consider gaze as a factor affecting viewing. The study by Borji et al. (2014), who tested the complementary effects of (low-level) saliency and (high-level) gaze in free viewing of scenes, is set along these same lines, but crucially, investigates the effects of gaze, an ‘overlooked attentional cue’ (see also Langton et al., 2006). In the current study, we add to this research by focusing on the high-level factors of gaze and semantic context, and using a task (visual search) that promotes the use of top-down information (Foulsham & Underwood, 2007). Specific models of visual search built also on evidence from the many studies that investigated effects of both low-level saliency and high-level semantic context but that used scenes that contained no people (Navalpakkam & Itti, 2005; Rao, Zelinsky, Hayhoe, & Ballard, 2002; Wolfe, 1994). Because people are commonly part of the visual environments of our everyday life, it is important to investigate how search occurs in such more ‘social’ scenes. Our results show that gaze has a key influence on viewing for visual search, and should thus be taken into account in visual search models, as well as in more broad models of visual attention. Moreover, they highlight the importance of computer vision models such as Recasens et al.’s (2015), that learn to extract gaze (and saliency) information from scenes.

These findings from studies using scenes are also relevant for the long-established research on gaze following. Such research reported repeatedly that people are faster at identifying targets that appear on the side of the screen where someone else is looking at (e.g., Friesen & Kingstone, 1998; Slessor et al., 2008), but most studies used the traditional gaze-cueing paradigm (e.g., Friesen & Kingstone, 1998), where a face is presented in isolation (though see Freeth et al., 2010, and Riby et al., 2013, who used scenes, but did not employ a visual search paradigm; Doherty, Patai Duta, Nobre, & Scerif, 2017, for effects of human distractors on search for objects in scenes). A notable exception is the study by Kuhn et al. (2018), who investigated gaze effects using real-world scenes and a (superimposed line) target detection task (Experiment 2). Like Kuhn and colleagues, we provide evidence for gaze following also in a task involving target detection, but in the more naturalistic context of scenes, addressing limitations of the decontextualized gaze cueing paradigm, which has been criticised for its artificial nature (e.g., Risko et al., 2012; Skarratt et al., 2012). Real-world scenes provide a richer visual context, which is important in the study of gaze effects. As noted by Frischen, Bayliss, and Tipper (2007), gaze processing is context sensitive: Following gaze directed to an object or to an empty space activates different brain regions responsive to perceived gaze direction (Pelphrey, Singerman, Allison, & McCarthy, 2003), and gaze cueing is stronger toward whole than scrambled objects (Bayliss & Tipper, 2005). The background and objects in a scene make it meaningful, and that meaning is one well-studied high-level factor affecting eye movements during viewing.

Research on scene-object inconsistencies builds on the idea that perceiving a scene involves the creation of a scene-specific schema or frame (Biederman, 1981; Biederman, Mezzanotte, & Rabinowitz, 1982; Friedman, 1979), based on which observers create expectations about the content and layout of the ‘pieces’ composing that scene. Indeed, schema aid the recognition of meaningful spatial relations among objects (Mandler & Johnson, 1976), and that is why objects are more difficult to identify in jumbled scenes (where pieces of it are cut and rearranged in a non-meaningful way; Biederman, 1972), and objects that violate the gist of the scene are more effortful to process (e.g., De Graef et al., 1990). Our experiment manipulated, alongside gaze, the semantics of the target object, which could be consistent or inconsistent with the gist of the scene. The main effects of consistency we found in measures of early visual search indicate that, overall, objects that were semantically consistent with the gist of the scene were selected first, compared to scene-inconsistent objects (in agreement with some, e.g., Võ & Henderson, 2011, but not other, e.g., Borges et al., 2019, previous findings; we return to this issue below). Yet, these effects were not observed equally across the different gaze conditions for each group. For older adults, search was faster (as reflected in time to first fixate) for consistent compared to inconsistent targets both when the targets were gazed at and when they were ignored by the actor in the scene. Younger adults, however, displayed this advantageous effect of consistency when the target object was gazed at by the actor, but not in the non-gazed-at conditions. This seems surprising if we consider prior studies reporting effects of semantic consistency (e.g., Henderson et al., 1999; Võ & Henderson, 2011): These studies tested younger adults using scenes containing no people and, therefore, no gaze cues, which we might think would be replicated in our non-gazed conditions. We should note, however, that our scenes cannot be directly compared with those of such previous studies. In the current stimuli, when the target object was not being looked at, the actor was instead looking at a distractor object (see Fig. 1A and D). We hypothesised that, in such conditions, the gazed-at distractor object would attract initial fixations of younger adults, which might have reduced the dependence of the subsequent search for the target on semantic context. In support of this hypothesis, we found that, where the distractor object was looked at by the actor in the scene, younger adults were more likely to fixate upon it early, regardless of target consistency. In contrast, older adults were not, unless the competing target object was inconsistent.

Our conditions where the actor looked at the distractor object, while the target object was consistent with the context, constitute the visual environment where gaze and consistency *compete* as cues guiding eye movements. Prioritizing *gaze* would result in looking at the (gazed-at) distractor, whereas prioritizing semantic *context* would result in observers looking at the (consistent) target. These were the patterns observed in the analyses of the probabilities of initial fixations, for younger and older adults, respectively, suggesting that younger adults prioritized gaze cues and older adults were more dependent on semantic context, to guide their initial eye movements on the scene. Such a pattern is in line with prior evidence for a stronger reliance on context to guide search in scenes by older adults (Borges et al., 2019; Neider & Kramer, 2011), and with the findings from several studies showing that younger adults follow gaze more than older adults in the traditional gaze-cueing paradigm (Kuhn et al., 2015; Slessor et al., 2008, 2010, 2016).

Our findings thus suggest that younger and older people show different integration of gaze and consistency information to guide initial attention. They emphasise the importance of encompassing gaze as a potential factor guiding scene viewing in models of visual inspection, and of considering that the relative weightings of different factors are likely to vary across the lifespan. Models such as Torralba et al.’s (2006), which combine different sources of information probabilistically, are suited for further extension to include these new factors. In such a model, each location in a scene is ranked a probability of containing a specific target given properties of that location concerning visual salience and scene context. Likewise, locations that are gazed at by people in the scene should be assigned a higher probability of target presence, compared to ignored locations, as proposed already by Recasens et al. (2015). Importantly, the probabilities given context and gaze would have different weights for younger and older adults. Continuing with Torralba et al.’s (2006) model, and its assumption that the ‘target probability’ map of the scene is computed very quickly and before the first saccade is deployed, we might expect that, for younger adults, the stronger weight of gaze would override any consistency effect in the non-gazed-at conditions. In the same conditions, for older adults, consistency might be weighted more than gaze.

Models of visual inspection should also take into consideration the different phases of search. The age differences we observed were in the initial phases of search, but not in later processes indexed by the total time fixating the target and the time to answer. In these measures, we found only main effects of gaze and consistency. We found that, for both groups, inconsistent targets were looked at for longer and elicited longer answer times than consistent objects, congruent with many prior studies, and the idea that unexpected objects in a scene are more effortful to process (e.g., Borges et al., 2019; De Graef et al., 1990; Henderson et al., 1999; Loftus & Mackworth, 1978; Underwood et al., 2008; Võ & Henderson, 2009). Likewise, both groups took more time to inspect and to answer to targets that were not gazed at compared to the ones attended by the actor. This result stands in contrast with the studies by Castelhano et al. (2007), Borji et al. (2014) and Kuhn et al. (2018, Experiment 1), where viewers fixated more gazed-at compared to non-gazed-at objects. Most likely, this difference results from the use of different tasks. In our search task, participants looked for a specific target object that is part of the scene. A target that is not being gazed at violates the expectation that gaze is a reliable cue to guide search, and it may thus give rise to an additional need to confirm its identity, such as scene-inconsistent targets do. Maybe more important, our non-gazed-at target has to be attended by viewers, as they are searching for it, whereas there is no reason why participants who viewed scenes in the studies by Castelhano et al., Borji et al. and Kuhn et al. would look more at any non-gazed-at region or object, compared to a region or object gazed at by the person in the scene.

In this respect, our task resembles more the typical gaze-cueing paradigm, where participants are asked to respond to a target that can appear at one or another side of a centrally presented face with the gaze averted left or right. The main finding is that participants take longer to answer to targets appearing at the opposite location, compared to the location where the gaze cue was directed to. Our results, showing longer times to respond to non-gazed-at, compared to gazed-at targets, are consistent with gaze-cueing studies. They do not replicate, however, results showing that gaze cueing response time effects are of smaller magnitude in old than younger people (e.g., Slessor et al., 2008). It is possible that, in more naturalistic tasks that use complex scenes and not faces in isolation, age differences in gaze cueing are evident in early but not later stages of visual processing. It has been suggested that such age differences might be related to gaze cueing effects peaking earlier for younger than older adults (Deroche, Castanier, Perrot, & Hartley, 2016), and this could have been reflected, in the current experiment, only on the initial eye movements. Further research would, however, be needed to test this hypothesis as, to our knowledge, there are to date no other studies investigating age differences in gaze following in the context of real-world scenes that could capture different points on the time-course of the effects.

Related to this, the current findings also speak to the question of whether gaze following is an automatic biological response to an eye-gaze cue, or whether it might reflect higher-level processing mechanisms. The use of scenes in the investigation of gaze following allows for manipulation of both attentional capture by gaze cues, and higher-level aspects of the scenes: If gaze following is an automatic process, it should be found irrespective of the semantic consistency of the target object and not be modulated by it. The interaction found for younger participants – consistency modulated the time to first fixate gazed-at targets but not non-gazed-at targets – suggests that higher-level factors such as scene context may modulate gaze-cuing effects, arguing against a purely automatic effect of gaze cues in complex social scenes, even when such effects occur fast (see Kuhn et al., 2018, and references therein for different concepts of automaticity in gaze following). In other words, our results dispute the view of gaze following as a purely bottom-up, low-level perceptual process (e.g., Driver et al., 1999).

We should note, however, that our findings as indexed by three-way interactions between group, gaze and consistency on time to first fixation and initial probabilities of fixation should be interpreted with caution. In our experiment each of the 64 participants provided eight repeated measures for each of the four conditions crossing gaze and consistency, which may not give our study enough power for reliably interpret interactions (e.g., Brysbaert, 2019; Brysbaert & Stevens, 2018). While we have employed mixed effects modelling, where within-participant variance across repeated measures is accommodated, thereby avoiding underestimation of standard errors and consequent Type 1 error inflation (Kliegl et al., 2010; Muth et al., 2016), we nevertheless acknowledge the potential fragility of these findings, which call for further research that attempts to replicate our study.

A final issue concerns the effects of consistency: Here we found that consistent items were located sooner than inconsistent. It is an open controversy whether scene-consistent objects attract early visual attention (i.e., the initial fixations) more or less than scene-inconsistent objects (see Wu, Wang, & Pomplun, 2014, for a review). In visual search, Võ and Henderson (2011) reported a consistency advantage, whereas Borges et al. (2019) found an inconsistency advantage. A possible explanation for the differences between our study and the one by Borges and colleagues concerns the priming manipulation implemented in the latter. In their study, participants were primed before each scene by a related scene in one condition, or by an unrelated scene in another. This indicates that task demands may strongly influence search strategies, and additional research is still needed to further clarify in which conditions consistent objects may not be prioritized (see, for further discussion, e.g., Spotorno & Tatler, 2017).

Although our study is considerably more ecologically valid than previous studies of age differences in gaze following, there are a number of limitations of this study as a model of real-world visual behaviour. The scenes were constructed to be very carefully controlled in terms of the age of the actors, the position and nature of objects, and the background scenes used. It would be useful to also look at age differences in natural gaze behaviour when looking at more varied and rich scenes containing people and using less constrained tasks such as free viewing, where more inspection time may allow other non-automatic processes to occur, such as the computation of the viewing perspective of gazers in the scenes (Kuhn et al., 2018). Also, the current study focused on fixed-position eye-tracking while viewing 2D scenes on a computer. To really understand how people of different ages use visual cues such as eye gaze when interacting in real life, mobile eye-tracking could be used. Finally, given the known effects of low-level visual properties on scene inspection, it would be important to further investigate how visual salience interacts with the higher-level gaze and context factors to achieve a more complete understanding of visual search in scenes.

## Conclusion

Our study extends current understanding of the influence of gaze direction as a cue to visual search, in addition to the influence of semantic factors and low-level visual features. By showing that consistency interacted with gaze, our study corroborates previous evidence for a complex interplay between sources of information determining scene viewing. Finally, our study highlights the importance of accounting for age-differences in viewing strategies. While both younger and older participants were influenced by gaze and context cues in visual search, the balance between the two was different: Younger adults relied more on gaze cues to guide early visual attention, while older adults were more influenced by semantic context. Overall, these results show the importance of using rich visual scenes to understand the interplay of viewer characteristics such as age with scene properties such as context and gaze cues to influence visual attention.

The data and materials for this experiment are available as Online Supplementary Material. This experiment was not preregistered.

## Electronic Supplementary Material

ESM 1 (XLSX 359 kb)

ESM 2 (ZIP 10.7 MB)

## Footnotes

1 We did not rotate the identity of the photographed actor across lists, but only assured that each scene had, for each condition crossing gaze and consistency, a version with a younger and a version with an older actor. Each participant saw the same actor twice in the experiment, but in a different scene and experimental condition.

2 Given the loss of some data and the difference in number of participants in the two groups, there were not the same number of observations in each two levels of a variable. The centring of factors takes this imbalance into account and thus attributes coefficients that may diverge from the -0.5 vs. 0.5 that would be attributed otherwise. For example, we had 982 observations from the young group and 869 observations from the older group, corresponding to 0.47 and 0.53 of the data, which is reflected in the coefficients for group (old, -.53; young, .47).

3 The first fixation is where the eyes were fixating at the scene onset and, therefore, before the first eye movement (saccade) performed after the scene presentation. The second fixation is where the eyes landed after the first saccade, and thus it is the first index of a fixation driven by viewing the scene. The third fixation is the fixation after the second saccade, and so forth, until the tenth fixation, which is the endpoint of the ninth saccade.
