# Supplementary figures and images for: The interplay between gaze and consistency in scene viewing: Evidence from visual search by young and older adults (part 1 of 2)
Source: Atten Percept Psychophys. 2021 Mar 21;83(5):1954–70. doi: 10.3758/s13414-021-02242-z (PMC8213592; doi:10.3758/s13414-021-02242-z)

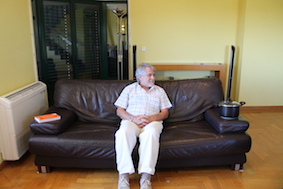

Supplement: Supplementary file 2 — (ZIP 10.7 MB) [file 13414_2021_2242_MOESM2_ESM.zip › miniatureImages/7_pot_bigSofa_I_gazed_R_LS_old_AC.JPG]

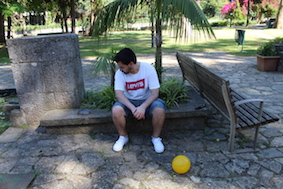

Supplement: Supplementary file 2 — (ZIP 10.7 MB) [file 13414_2021_2242_MOESM2_ESM.zip › miniatureImages/21_glassesCase_pulpit_C_gazed_L_LS_young_JJ.JPG]

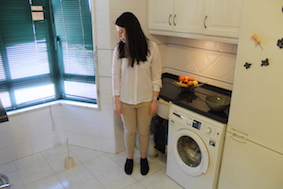

Supplement: Supplementary file 2 — (ZIP 10.7 MB) [file 13414_2021_2242_MOESM2_ESM.zip › miniatureImages/2_toiletBrush_kitchenFloor_I_gazed_L_LS_young_RC.JPG]

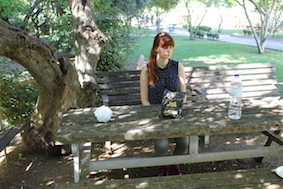

Supplement: Supplementary file 2 — (ZIP 10.7 MB) [file 13414_2021_2242_MOESM2_ESM.zip › miniatureImages/20_bathSponge_picnicTable_I_nongazed_L_LS_young_JM.JPG]

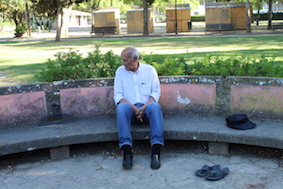

Supplement: Supplementary file 2 — (ZIP 10.7 MB) [file 13414_2021_2242_MOESM2_ESM.zip › miniatureImages/19_clothesHanger_pinkBench_I_gazed_L_LS_old_VS.JPG]

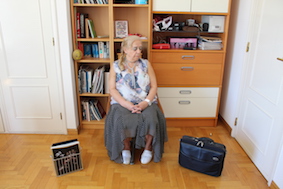

Supplement: Supplementary file 2 — (ZIP 10.7 MB) [file 13414_2021_2242_MOESM2_ESM.zip › miniatureImages/15_magazineHolder_officeFloor_C_nongazed_L_LS_old_CC.JPG]

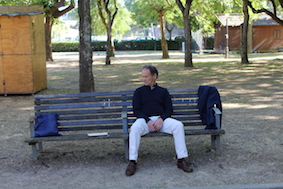

Supplement: Supplementary file 2 — (ZIP 10.7 MB) [file 13414_2021_2242_MOESM2_ESM.zip › miniatureImages/17_coat_woodBench_C_nongazed_R_LS_old_MF.JPG]

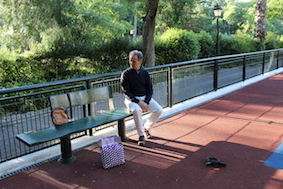

Supplement: Supplementary file 2 — (ZIP 10.7 MB) [file 13414_2021_2242_MOESM2_ESM.zip › miniatureImages/28_skippingRope_playground2_C_nongazed_R_LS_old_MF.JPG]

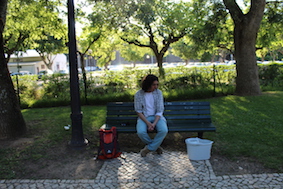

Supplement: Supplementary file 2 — (ZIP 10.7 MB) [file 13414_2021_2242_MOESM2_ESM.zip › miniatureImages/32_bucket_greenWoodBench_I_gazed_R_LS_young_MC.JPG]

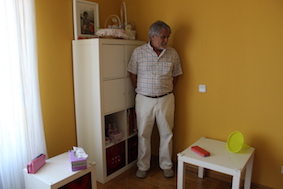

Supplement: Supplementary file 2 — (ZIP 10.7 MB) [file 13414_2021_2242_MOESM2_ESM.zip › miniatureImages/6_funnel_kidsBedroom_I_gazed_R_LS_old_AC.JPG]

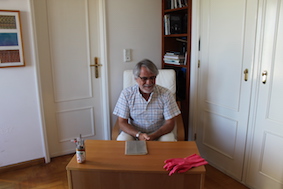

Supplement: Supplementary file 2 — (ZIP 10.7 MB) [file 13414_2021_2242_MOESM2_ESM.zip › miniatureImages/14_rubberGloves_officeTable_I_gazed_R_LS_old_AC.JPG]

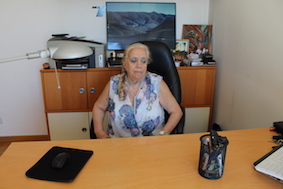

Supplement: Supplementary file 2 — (ZIP 10.7 MB) [file 13414_2021_2242_MOESM2_ESM.zip › miniatureImages/16_computerMouse_officeDesk_C_nongazed_L_LS_old_CC.JPG]

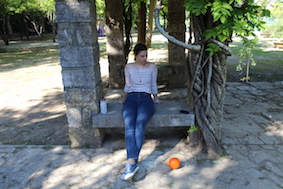

Supplement: Supplementary file 2 — (ZIP 10.7 MB) [file 13414_2021_2242_MOESM2_ESM.zip › miniatureImages/23_waterBottle_pilarsBench_C_nongazed_L_LS_young_DS.JPG]

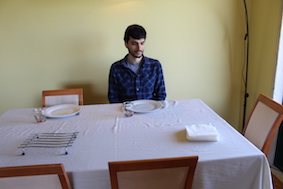

Supplement: Supplementary file 2 — (ZIP 10.7 MB) [file 13414_2021_2242_MOESM2_ESM.zip › miniatureImages/10_napkins_dinningTable_C_gazed_R_LS_young_MX.JPG]

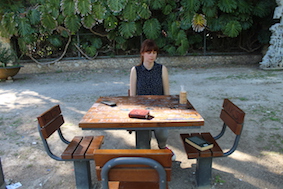

Supplement: Supplementary file 2 — (ZIP 10.7 MB) [file 13414_2021_2242_MOESM2_ESM.zip › miniatureImages/22_TVcontrol_table_I_nongazed_L_LS_young_JM.JPG]

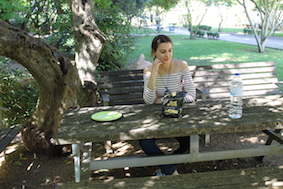

Supplement: Supplementary file 2 — (ZIP 10.7 MB) [file 13414_2021_2242_MOESM2_ESM.zip › miniatureImages/20_plate_picnicTable_C_nongazed_L_LS_young_DS.JPG]

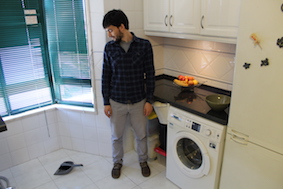

Supplement: Supplementary file 2 — (ZIP 10.7 MB) [file 13414_2021_2242_MOESM2_ESM.zip › miniatureImages/2_dustpanBrush_kitchenFloor_C_gazed_L_LS_young_MX.JPG]

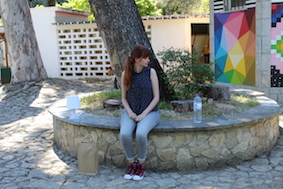

Supplement: Supplementary file 2 — (ZIP 10.7 MB) [file 13414_2021_2242_MOESM2_ESM.zip › miniatureImages/18_lamp_wallBench_I_nongazed_L_LS_young_JM.JPG]

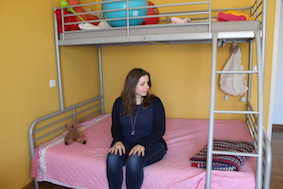

Supplement: Supplementary file 2 — (ZIP 10.7 MB) [file 13414_2021_2242_MOESM2_ESM.zip › miniatureImages/5_softToy_kidsBed_C_nongazed_L_LS_young_RP.JPG]

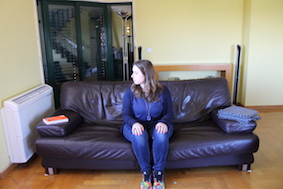

Supplement: Supplementary file 2 — (ZIP 10.7 MB) [file 13414_2021_2242_MOESM2_ESM.zip › miniatureImages/7_throw_bigSofa_C_nongazed_R_LS_young_RP.JPG]

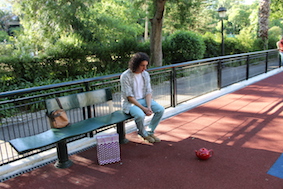

Supplement: Supplementary file 2 — (ZIP 10.7 MB) [file 13414_2021_2242_MOESM2_ESM.zip › miniatureImages/28_teapot_playground2_I_gazed_R_LS_young_MC.JPG]

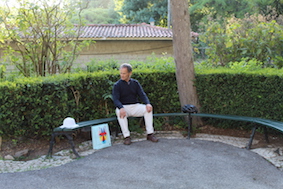

Supplement: Supplementary file 2 — (ZIP 10.7 MB) [file 13414_2021_2242_MOESM2_ESM.zip › miniatureImages/31_helmet_roundGreenBench_C_nongazed_R_LS_old_MF.JPG]

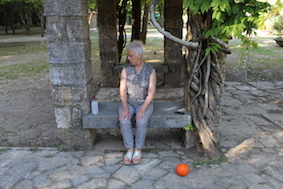

Supplement: Supplementary file 2 — (ZIP 10.7 MB) [file 13414_2021_2242_MOESM2_ESM.zip › miniatureImages/23_waterBottle_pilarsBench_C_gazed_L_LS_old_CF.JPG]

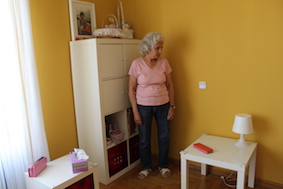

Supplement: Supplementary file 2 — (ZIP 10.7 MB) [file 13414_2021_2242_MOESM2_ESM.zip › miniatureImages/6_lamp_kidsBedroom_C_gazed_R_LS_old_AF.JPG]

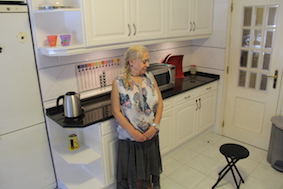

Supplement: Supplementary file 2 — (ZIP 10.7 MB) [file 13414_2021_2242_MOESM2_ESM.zip › miniatureImages/3_kettle_kitchenCounter_C_nongazed_L_LS_old_CC.JPG]

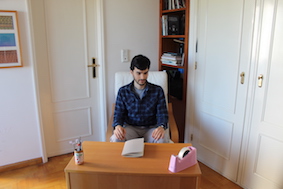

Supplement: Supplementary file 2 — (ZIP 10.7 MB) [file 13414_2021_2242_MOESM2_ESM.zip › miniatureImages/14_tapDispenser_officeTable_C_gazed_R_LS_young_MX.JPG]

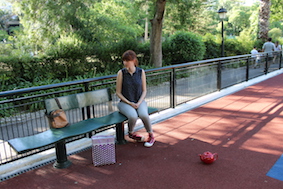

Supplement: Supplementary file 2 — (ZIP 10.7 MB) [file 13414_2021_2242_MOESM2_ESM.zip › miniatureImages/28_teapot_playground2_I_nongazed_R_LS_young_JM.JPG]

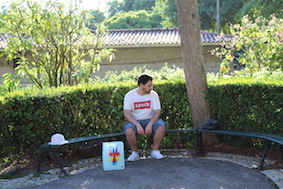

Supplement: Supplementary file 2 — (ZIP 10.7 MB) [file 13414_2021_2242_MOESM2_ESM.zip › miniatureImages/31_helmet_roundGreenBench_C_gazed_R_LS_young_JJ.JPG]

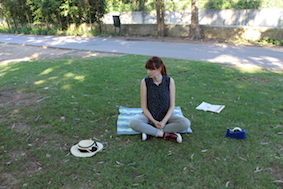

Supplement: Supplementary file 2 — (ZIP 10.7 MB) [file 13414_2021_2242_MOESM2_ESM.zip › miniatureImages/25_tapDispender_grass_I_nongazed_R_LS_young_JM.JPG]

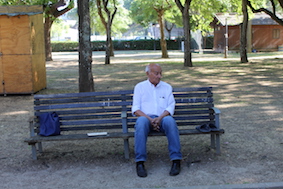

Supplement: Supplementary file 2 — (ZIP 10.7 MB) [file 13414_2021_2242_MOESM2_ESM.zip › miniatureImages/17_sleepers_woodBench_I_gazed_R_LS_old_VS.JPG]

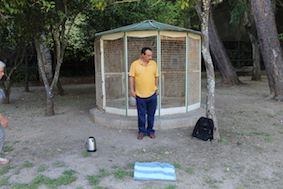

Supplement: Supplementary file 2 — (ZIP 10.7 MB) [file 13414_2021_2242_MOESM2_ESM.zip › miniatureImages/26_kettle_birdsCage_I_nongazed_L_LS_old_T.JPG]

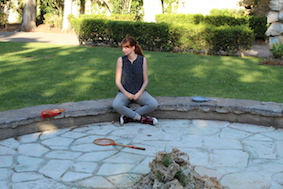

Supplement: Supplementary file 2 — (ZIP 10.7 MB) [file 13414_2021_2242_MOESM2_ESM.zip › miniatureImages/30_fryingPan_lake_I_nongazed_R_LS_young_JM.JPG]

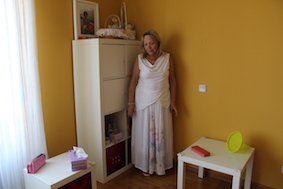

Supplement: Supplementary file 2 — (ZIP 10.7 MB) [file 13414_2021_2242_MOESM2_ESM.zip › miniatureImages/6_funnel_kidsBedroom_I_nongazed_R_LS_old_MM.JPG]

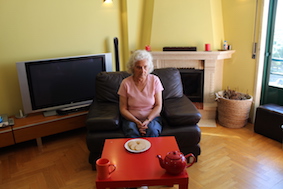

Supplement: Supplementary file 2 — (ZIP 10.7 MB) [file 13414_2021_2242_MOESM2_ESM.zip › miniatureImages/8_teapot_smallSofaTV_C_gazed_R_LS_old_AF.JPG]

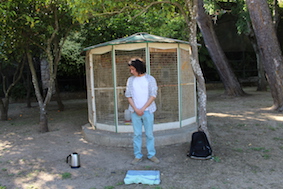

Supplement: Supplementary file 2 — (ZIP 10.7 MB) [file 13414_2021_2242_MOESM2_ESM.zip › miniatureImages/26_kettle_birdsCage_I_gazed_L_LS_young_MC.JPG]

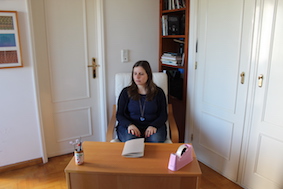

Supplement: Supplementary file 2 — (ZIP 10.7 MB) [file 13414_2021_2242_MOESM2_ESM.zip › miniatureImages/14_tapDispenser_officeTable_C_nongazed_R_LS_young_RP.JPG]

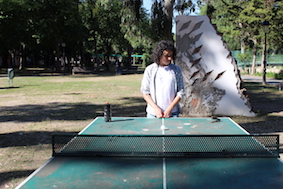

Supplement: Supplementary file 2 — (ZIP 10.7 MB) [file 13414_2021_2242_MOESM2_ESM.zip › miniatureImages/24_stapler_pingpongTable_I_gazed_R_LS_young_MC.JPG]

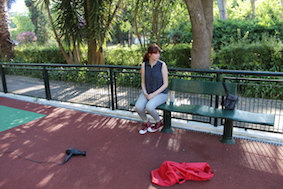

Supplement: Supplementary file 2 — (ZIP 10.7 MB) [file 13414_2021_2242_MOESM2_ESM.zip › miniatureImages/27_hairdryer_playground1_I_nongazed_L_LS_young_JM.JPG]

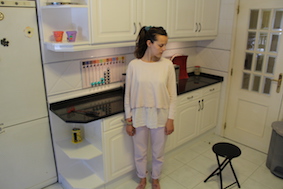

Supplement: Supplementary file 2 — (ZIP 10.7 MB) [file 13414_2021_2242_MOESM2_ESM.zip › miniatureImages/3_rake_kitchenCounter_I_nongazed_L_LS_young_MC.JPG]

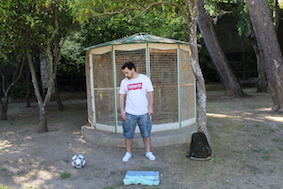

Supplement: Supplementary file 2 — (ZIP 10.7 MB) [file 13414_2021_2242_MOESM2_ESM.zip › miniatureImages/26_ball_birdsCage_C_gazed_L_LS_young_JJ.JPG]

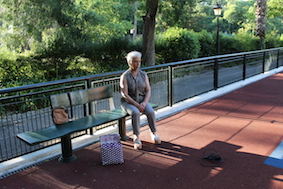

Supplement: Supplementary file 2 — (ZIP 10.7 MB) [file 13414_2021_2242_MOESM2_ESM.zip › miniatureImages/28_skippingRope_playground2_C_gazed_R_LS_old_CF.JPG]

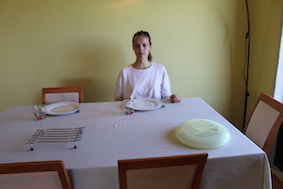

Supplement: Supplementary file 2 — (ZIP 10.7 MB) [file 13414_2021_2242_MOESM2_ESM.zip › miniatureImages/10_frisbee_dinningTable_I_nongazed_R_LS_young_MC.JPG]

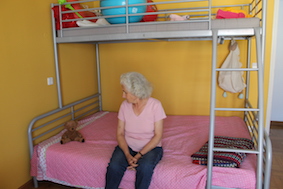

Supplement: Supplementary file 2 — (ZIP 10.7 MB) [file 13414_2021_2242_MOESM2_ESM.zip › miniatureImages/5_softToy_kidsBed_C_gazed_L_LS_old_AF.JPG]

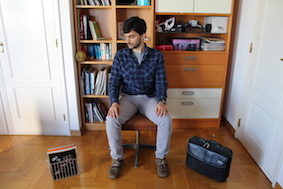

Supplement: Supplementary file 2 — (ZIP 10.7 MB) [file 13414_2021_2242_MOESM2_ESM.zip › miniatureImages/15_magazineHolder_officeFloor_C_gazed_L_LS_young_MX.JPG]

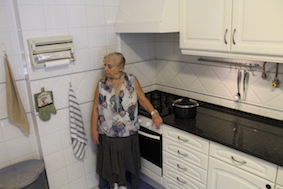

Supplement: Supplementary file 2 — (ZIP 10.7 MB) [file 13414_2021_2242_MOESM2_ESM.zip › miniatureImages/4_pot_kitchenStove_C_nongazed_R_LS_old_CC.JPG]

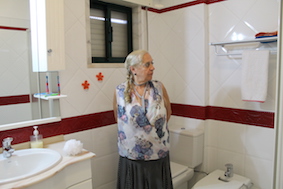

Supplement: Supplementary file 2 — (ZIP 10.7 MB) [file 13414_2021_2242_MOESM2_ESM.zip › miniatureImages/12_bathSponge_bathroom_C_nongazed_L_LS_old_CC.JPG]

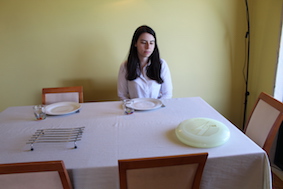

Supplement: Supplementary file 2 — (ZIP 10.7 MB) [file 13414_2021_2242_MOESM2_ESM.zip › miniatureImages/10_frisbee_dinningTable_I_gazed_R_LS_young_RC.JPG]

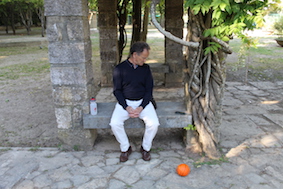

Supplement: Supplementary file 2 — (ZIP 10.7 MB) [file 13414_2021_2242_MOESM2_ESM.zip › miniatureImages/23_waterBottle_pilarsBench_C_nongazed_L_LS_old_MF.JPG]

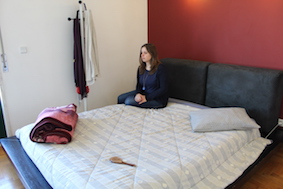

Supplement: Supplementary file 2 — (ZIP 10.7 MB) [file 13414_2021_2242_MOESM2_ESM.zip › miniatureImages/13_pillow_bedroom_C_nongazed_R_LS_young_RP.JPG]

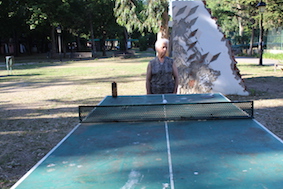

Supplement: Supplementary file 2 — (ZIP 10.7 MB) [file 13414_2021_2242_MOESM2_ESM.zip › miniatureImages/24_pingpongBat_pingpongTable_C_gazed_R_LS_old_CF.JPG]

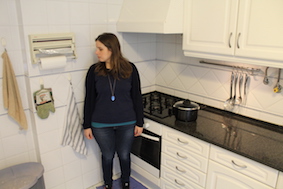

Supplement: Supplementary file 2 — (ZIP 10.7 MB) [file 13414_2021_2242_MOESM2_ESM.zip › miniatureImages/4_pot_kitchenStove_C_nongazed_R_LS_young_RP.JPG]

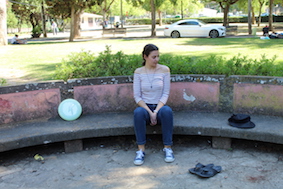

Supplement: Supplementary file 2 — (ZIP 10.7 MB) [file 13414_2021_2242_MOESM2_ESM.zip › miniatureImages/19_frisbee_pinkBench_C_nongazed_L_LS_young_DS.JPG]

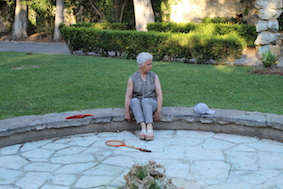

Supplement: Supplementary file 2 — (ZIP 10.7 MB) [file 13414_2021_2242_MOESM2_ESM.zip › miniatureImages/30_cap_lake_C_gazed_R_LS_old_CF.JPG]

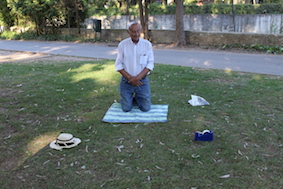

Supplement: Supplementary file 2 — (ZIP 10.7 MB) [file 13414_2021_2242_MOESM2_ESM.zip › miniatureImages/25_tapDispender_grass_I_gazed_R_LS_old_VS.JPG]

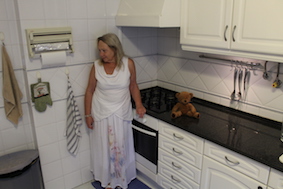

Supplement: Supplementary file 2 — (ZIP 10.7 MB) [file 13414_2021_2242_MOESM2_ESM.zip › miniatureImages/4_softToy_kitchenStove_I_nongazed_R_LS_old_MM.JPG]

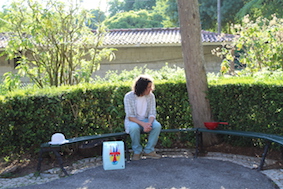

Supplement: Supplementary file 2 — (ZIP 10.7 MB) [file 13414_2021_2242_MOESM2_ESM.zip › miniatureImages/31_colander_roundGreenBench_I_gazed_R_LS_young_MC.JPG]

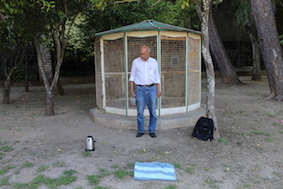

Supplement: Supplementary file 2 — (ZIP 10.7 MB) [file 13414_2021_2242_MOESM2_ESM.zip › miniatureImages/26_kettle_birdsCage_I_gazed_L_LS_old_VS.JPG]

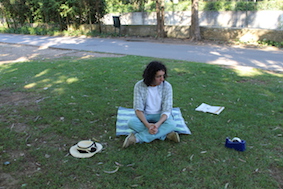

Supplement: Supplementary file 2 — (ZIP 10.7 MB) [file 13414_2021_2242_MOESM2_ESM.zip › miniatureImages/25_tapDispender_grass_I_gazed_R_LS_young_MC.JPG]

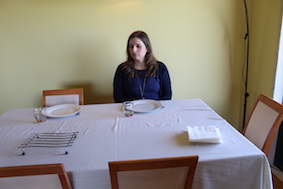

Supplement: Supplementary file 2 — (ZIP 10.7 MB) [file 13414_2021_2242_MOESM2_ESM.zip › miniatureImages/10_napkins_dinningTable_C_nongazed_R_LS_young_RP.JPG]

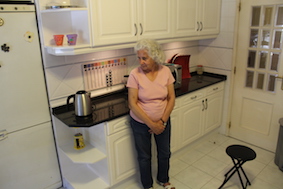

Supplement: Supplementary file 2 — (ZIP 10.7 MB) [file 13414_2021_2242_MOESM2_ESM.zip › miniatureImages/3_kettle_kitchenCounter_C_gazed_L_LS_old_AF.JPG]

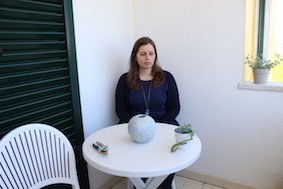

Supplement: Supplementary file 2 — (ZIP 10.7 MB) [file 13414_2021_2242_MOESM2_ESM.zip › miniatureImages/11_secateurs_balcony_C_nongazed_L_LS_young_RP.JPG]

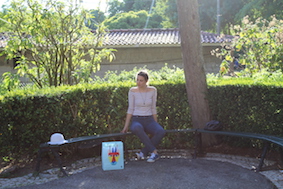

Supplement: Supplementary file 2 — (ZIP 10.7 MB) [file 13414_2021_2242_MOESM2_ESM.zip › miniatureImages/31_helmet_roundGreenBench_C_nongazed_R_LS_young_DS.JPG]

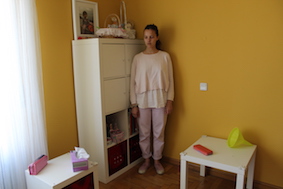

Supplement: Supplementary file 2 — (ZIP 10.7 MB) [file 13414_2021_2242_MOESM2_ESM.zip › miniatureImages/6_funnel_kidsBedroom_I_nongazed_R_LS_young_MC.JPG]

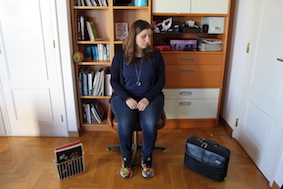

Supplement: Supplementary file 2 — (ZIP 10.7 MB) [file 13414_2021_2242_MOESM2_ESM.zip › miniatureImages/15_magazineHolder_officeFloor_C_nongazed_L_LS_young_RP.JPG]

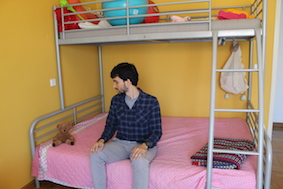

Supplement: Supplementary file 2 — (ZIP 10.7 MB) [file 13414_2021_2242_MOESM2_ESM.zip › miniatureImages/5_softToy_kidsBed_C_gazed_L_LS_young_MX.JPG]

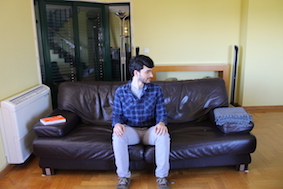

Supplement: Supplementary file 2 — (ZIP 10.7 MB) [file 13414_2021_2242_MOESM2_ESM.zip › miniatureImages/7_throw_bigSofa_C_gazed_R_LS_young_MX.JPG]

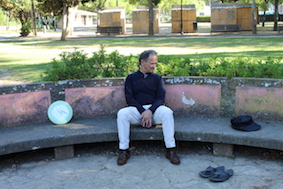

Supplement: Supplementary file 2 — (ZIP 10.7 MB) [file 13414_2021_2242_MOESM2_ESM.zip › miniatureImages/19_frisbee_pinkBench_C_nongazed_L_LS_old_MF.JPG]

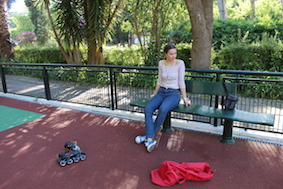

Supplement: Supplementary file 2 — (ZIP 10.7 MB) [file 13414_2021_2242_MOESM2_ESM.zip › miniatureImages/27_rollerblades_playground1_C_nongazed_L_LS_young_DS.JPG]

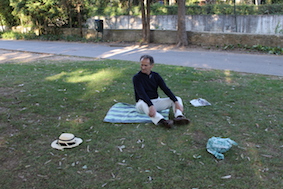

Supplement: Supplementary file 2 — (ZIP 10.7 MB) [file 13414_2021_2242_MOESM2_ESM.zip › miniatureImages/25_scarf_grass_C_nongazed_R_LS_old_MF.JPG]

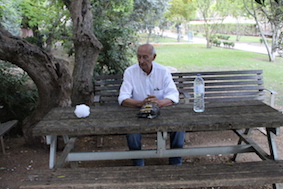

Supplement: Supplementary file 2 — (ZIP 10.7 MB) [file 13414_2021_2242_MOESM2_ESM.zip › miniatureImages/20_bathSponge_picnicTable_I_gazed_L_LS_old_VS.JPG]

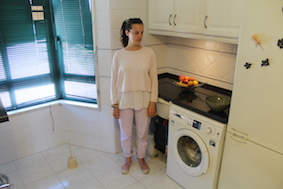

Supplement: Supplementary file 2 — (ZIP 10.7 MB) [file 13414_2021_2242_MOESM2_ESM.zip › miniatureImages/2_toiletBrush_kitchenFloor_I_nongazed_L_LS_young_MC.JPG]

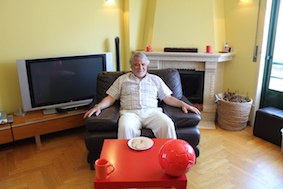

Supplement: Supplementary file 2 — (ZIP 10.7 MB) [file 13414_2021_2242_MOESM2_ESM.zip › miniatureImages/8_ball_smallSofaTV_I_gazed_R_LS_old_AC.JPG]

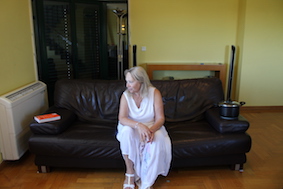

Supplement: Supplementary file 2 — (ZIP 10.7 MB) [file 13414_2021_2242_MOESM2_ESM.zip › miniatureImages/7_pot_bigSofa_I_nongazed_R_LS_old_MM.JPG]

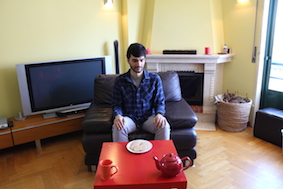

Supplement: Supplementary file 2 — (ZIP 10.7 MB) [file 13414_2021_2242_MOESM2_ESM.zip › miniatureImages/8_teapot_smallSofaTV_C_gazed_R_LS_young_MX.JPG]

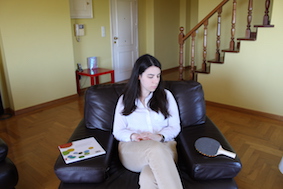

Supplement: Supplementary file 2 — (ZIP 10.7 MB) [file 13414_2021_2242_MOESM2_ESM.zip › miniatureImages/9_pingpongBat_smallSofaStairs_I_gazed_R_LS_young_RC.JPG]

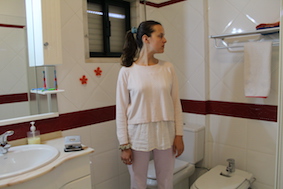

Supplement: Supplementary file 2 — (ZIP 10.7 MB) [file 13414_2021_2242_MOESM2_ESM.zip › miniatureImages/12_holePunch_bathroom_I_nongazed_L_LS_young_MC.JPG]

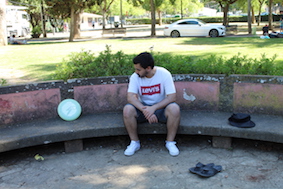

Supplement: Supplementary file 2 — (ZIP 10.7 MB) [file 13414_2021_2242_MOESM2_ESM.zip › miniatureImages/19_frisbee_pinkBench_C_gazed_L_LS_young_JJ.JPG]

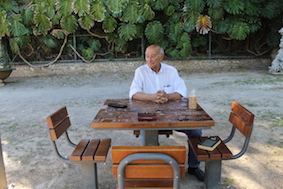

Supplement: Supplementary file 2 — (ZIP 10.7 MB) [file 13414_2021_2242_MOESM2_ESM.zip › miniatureImages/22_TVcontrol_table_I_gazed_L_LS_old_VS.JPG]

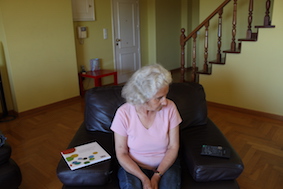

Supplement: Supplementary file 2 — (ZIP 10.7 MB) [file 13414_2021_2242_MOESM2_ESM.zip › miniatureImages/9_TVcontrol_smallSofaStairs_C_gazed_R_LS_old_AF.JPG]

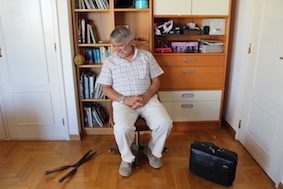

Supplement: Supplementary file 2 — (ZIP 10.7 MB) [file 13414_2021_2242_MOESM2_ESM.zip › miniatureImages/15_gardenShears_officeFloor_I_gazed_L_LS_old_AC.JPG]

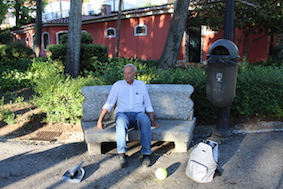

Supplement: Supplementary file 2 — (ZIP 10.7 MB) [file 13414_2021_2242_MOESM2_ESM.zip › miniatureImages/29_dustpanBrush_stoneBench_I_gazed_L_LS_old_VS.JPG]

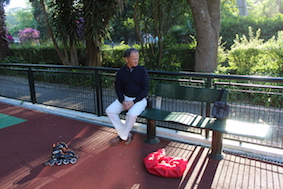

Supplement: Supplementary file 2 — (ZIP 10.7 MB) [file 13414_2021_2242_MOESM2_ESM.zip › miniatureImages/27_rollerblades_playground1_C_nongazed_L_LS_old_MF.JPG]

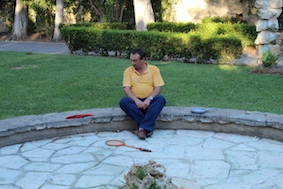

Supplement: Supplementary file 2 — (ZIP 10.7 MB) [file 13414_2021_2242_MOESM2_ESM.zip › miniatureImages/30_fryingPan_lake_I_nongazed_R_LS_old_T.JPG]

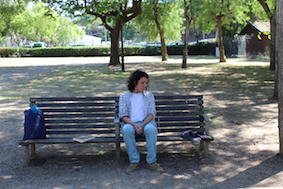

Supplement: Supplementary file 2 — (ZIP 10.7 MB) [file 13414_2021_2242_MOESM2_ESM.zip › miniatureImages/17_sleepers_woodBench_I_gazed_R_LS_young_MC.JPG]

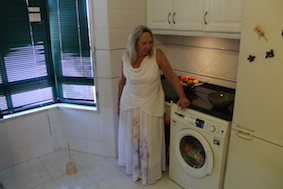

Supplement: Supplementary file 2 — (ZIP 10.7 MB) [file 13414_2021_2242_MOESM2_ESM.zip › miniatureImages/2_toiletBrush_kitchenFloor_I_nongazed_L_LS_old_MM.JPG]

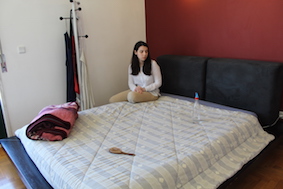

Supplement: Supplementary file 2 — (ZIP 10.7 MB) [file 13414_2021_2242_MOESM2_ESM.zip › miniatureImages/13_cleaningSpray_bedroom_I_gazed_R_LS_young_RC.JPG]

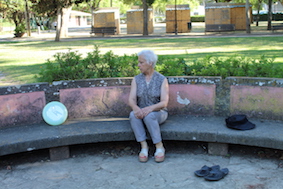

Supplement: Supplementary file 2 — (ZIP 10.7 MB) [file 13414_2021_2242_MOESM2_ESM.zip › miniatureImages/19_frisbee_pinkBench_C_gazed_L_LS_old_CF.JPG]

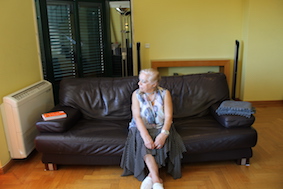

Supplement: Supplementary file 2 — (ZIP 10.7 MB) [file 13414_2021_2242_MOESM2_ESM.zip › miniatureImages/7_throw_bigSofa_C_nongazed_R_LS_old_CC.JPG]

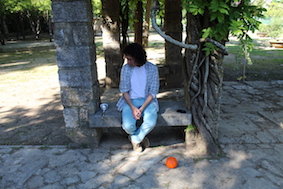

Supplement: Supplementary file 2 — (ZIP 10.7 MB) [file 13414_2021_2242_MOESM2_ESM.zip › miniatureImages/23_ladle_pilarsBench_I_gazed_L_LS_young_MC.JPG]

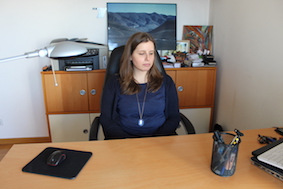

Supplement: Supplementary file 2 — (ZIP 10.7 MB) [file 13414_2021_2242_MOESM2_ESM.zip › miniatureImages/16_computerMouse_officeDesk_C_nongazed_L_LS_young_RP.JPG]

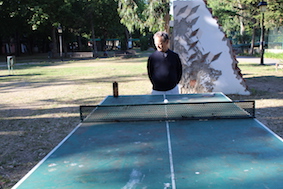

Supplement: Supplementary file 2 — (ZIP 10.7 MB) [file 13414_2021_2242_MOESM2_ESM.zip › miniatureImages/24_pingpongBat_pingpongTable_C_nongazed_R_LS_old_MF.JPG]

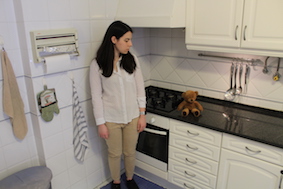

Supplement: Supplementary file 2 — (ZIP 10.7 MB) [file 13414_2021_2242_MOESM2_ESM.zip › miniatureImages/4_softToy_kitchenStove_I_gazed_R_LS_young_RC.JPG]

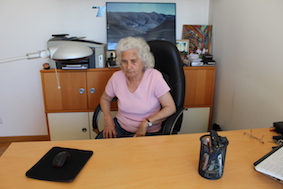

Supplement: Supplementary file 2 — (ZIP 10.7 MB) [file 13414_2021_2242_MOESM2_ESM.zip › miniatureImages/16_computerMouse_officeDesk_C_gazed_L_LS_old_AF.JPG]

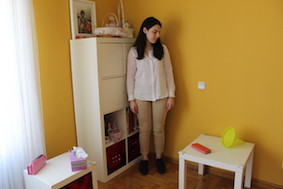

Supplement: Supplementary file 2 — (ZIP 10.7 MB) [file 13414_2021_2242_MOESM2_ESM.zip › miniatureImages/6_funnel_kidsBedroom_I_gazed_R_LS_young_RC.JPG]

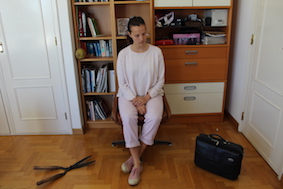

Supplement: Supplementary file 2 — (ZIP 10.7 MB) [file 13414_2021_2242_MOESM2_ESM.zip › miniatureImages/15_gardenShears_officeFloor_I_nongazed_L_LS_young_MC.JPG]

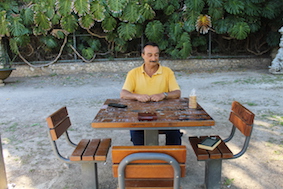

Supplement: Supplementary file 2 — (ZIP 10.7 MB) [file 13414_2021_2242_MOESM2_ESM.zip › miniatureImages/22_TVcontrol_table_I_nongazed_L_LS_old_T.JPG]

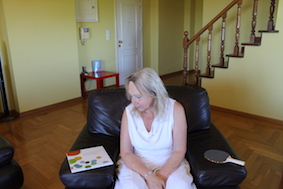

Supplement: Supplementary file 2 — (ZIP 10.7 MB) [file 13414_2021_2242_MOESM2_ESM.zip › miniatureImages/9_pingpongBat_smallSofaStairs_I_nongazed_R_LS_old_MM.JPG]

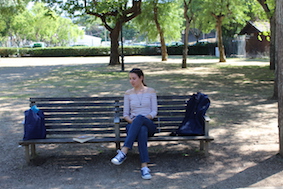

Supplement: Supplementary file 2 — (ZIP 10.7 MB) [file 13414_2021_2242_MOESM2_ESM.zip › miniatureImages/17_coat_woodBench_C_nongazed_R_LS_young_DS.JPG]

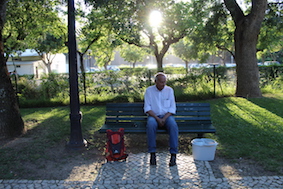

Supplement: Supplementary file 2 — (ZIP 10.7 MB) [file 13414_2021_2242_MOESM2_ESM.zip › miniatureImages/32_bucket_greenWoodBench_I_gazed_R_LS_old_VS.JPG]

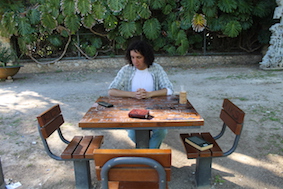

Supplement: Supplementary file 2 — (ZIP 10.7 MB) [file 13414_2021_2242_MOESM2_ESM.zip › miniatureImages/22_TVcontrol_table_I_gazed_L_LS_young_MC.JPG]
